# Challenge Level Contributes to the Efficacy of Treadmill Interventions after Stroke: A Systematic Review and Meta-Analysis

**DOI:** 10.3390/brainsci13121729

**Published:** 2023-12-18

**Authors:** Sharon Olsen, Gemma Alder, Usman Rashid, Emeline Gomes, Madeleine Aislabie, Fran Chee, Caitlin Smith, Brody Kean, Nicola Towersey, Nada Signal

**Affiliations:** 1 Rehabilitation Innovation Centre, Health and Rehabilitation Research Institute, Auckland University of Technology, Auckland 0627, New Zealand; gemma.alder@aut.ac.nz (G.A.); usman.rashid@aut.ac.nz (U.R.); emma.gomes@aut.ac.nz (E.G.); nicola.towersey@aut.ac.nz (N.T.); nada.signal@aut.ac.nz (N.S.); 2 Centre for Chiropractic Research, New Zealand College of Chiropractic, Auckland 1060, New Zealand

**Keywords:** treadmill training, dosage, dose, intensity, challenge, stroke, intervention reporting

## Abstract

Intervention parameters such as the challenge, amount, and dosage (challenge × amount) have the potential to alter the efficacy of rehabilitation interventions after stroke. This systematic review investigated the effect of intervention parameters of challenge, amount, and dosage on improvements in walking outcomes following treadmill training (TT) and comparison interventions in people with stroke. Randomized controlled trials were included if they: (i) investigated interventions of TT or bodyweight-supported TT (BWSTT); (ii) made comparisons with other physiotherapy interventions, other types of TT, or no intervention; (iii) studied people with stroke; (iv) reported sufficient data on challenge and amount parameters; and (v) measured walking speed or endurance. Completeness of reporting was evaluated using the TIDieR-Rehab checklist and risk of bias was assessed using the revised Cochrane risk-of-bias tool. The review included 26 studies; 15 studies compared TT or BWSTT with other physiotherapy interventions and 11 studies compared different types of TT. Meta-analyses provided evidence with low to moderate certainty that greater differences in challenge and dosage between treadmill and comparison physiotherapy interventions produced greater effects on walking endurance (*p* < 0.01). However, challenge and dosage did not influence walking speed outcomes. The analysis of intervention amount was limited by the lack of studies that manipulated the amount of intervention. Overall, the findings indicate that, after stroke, some of the efficacy of TT on walking endurance can be explained by the challenge level during training. This supports the implementation of TT at higher challenge levels in stroke rehabilitation practice.

## 1. Introduction

Stroke is the second leading cause of disability worldwide and a health and societal burden that is growing year by year [1]. The consequences for quality of life can be devastating due to the loss of autonomy and ability to participate in meaningful activities [2]. Rehabilitation goes some way to reduce this burden through task and context-specific training that facilitates the recovery of motor and cognitive function [3]. However, although significant recovery can occur during rehabilitation, many people with stroke continue to experience ongoing activity limitations after discharge from rehabilitation services [4,5]. Gait and balance are a particular priority [6], as most individuals still experience limited household or community mobility one year after stroke [7]. These ongoing limitations raise the question of whether current rehabilitation could be further optimized to improve stroke recovery.

Walking rehabilitation may be optimized through increased amounts of training. Task-specific training is known to promote neuroplasticity [8,9,10], with larger amounts correlated with improvements in walking ability, speed, and activities of daily living after stroke [11,12,13,14]. This understanding has prompted interest in treadmill training (TT) with or without bodyweight support (BWS) as a rehabilitation intervention, as it can enable large amounts of walking practice [15]. A 2017 systematic review demonstrated that TT or BWSTT significantly improved walking speed when applied ≥3 times weekly and significantly improved walking endurance when applied 5 times weekly for at least four weeks in people with stroke [16]. In addition to supporting larger amounts or repetitions of training, TT and BWSTT allow for convenient manipulation of the challenge of training.

Adaptation of the challenge of training can be achieved through manipulation of speed, incline, assistance offered, and percentage of BWS [15]. Challenge refers to how difficult, complex, or intensive a task is for an individual and the physical or mental effort required to complete the task [17]. However, the evidence concerning the optimal challenge point for TT is limited. This is due in part to the variability in definitions used across the literature to quantify the therapy administered. The terms “dose”, “intensity”, and “challenge”, for example, have been used interchangeably in many studies to describe the amount of therapy given and how much effort was required from the individual [13,14,16,17,18,19]. In this report, these different constructs are delineated and defined, where “amount” refers to the volume of therapy delivered, including the session duration, frequency per week, and length of the program [11], whereas “challenge” refers to how challenging the task is for the individual over a set period [17,19]. The combination of “amount” and “challenge” is referred to as “dosage” [20]. Across the rehabilitation literature, challenge has not been investigated to the same degree as the amount of intervention [16,21,22]. However, evidence from upper limb rehabilitation literature suggests that more challenging training results in improved outcomes following stroke [22,23], reinforcing the potential for using training challenge to optimize walking rehabilitation.

The existing literature on TT for individuals with stroke has focused on its optimal amount, as highlighted in a prior Cochrane systematic review [16]. In addition, an earlier review that considered challenge did not adequately address the comparative challenge levels relative to the neurological impairment of individuals in different intervention groups [24]. Thus, a comprehensive analysis considering the variables of challenge, amount, and dosage is lacking. This systematic review aimed to address this gap by thoroughly examining the effect of these parameters on walking outcomes for people with stroke. It investigated the effect of variations in training challenge, total amount, and overall dosage of TT and comparison interventions on improvements in walking outcomes in people with stroke. The findings of this research have the potential to inform the optimal training parameters for TT following stroke.

## 2. Materials and Methods

This systematic review was conducted in accordance with the Preferred Reporting Items for Systematic Reviews and Meta-Analyses (PRISMA) guidelines [25] and was registered in the PROSPERO database (n° CRD42020204289). 

### 2.1. Search Strategy 

Articles published prior to February 2017 were identified based on the 2017 Cochrane review of the efficacy of TT and BWSTT interventions for walking after stroke [16]. All studies in this review were screened for inclusion. For articles published after February 2017, studies were identified by replicating the search strategy used by Mehrholz et al. (2017) [16] in the online databases MEDLINE, AMED, CINAHL, SPORTDiscus, Scopus, and the Cochrane Central Register of Controlled Trials (CENTRAL). These databases were searched on 10 April 2022, with the start date limiter set to February 2017. The search terms for the database search were those utilized in the 2017 Cochrane review [16] for the respective databases except Scopus, which was adapted from the MEDLINE search terms and can be viewed in Supplementary File S1.

### 2.2. Study Screening and Eligibility Assessment

Titles and abstracts, as well as relevant full-text articles, were screened independently by pairs of reviewers (C.S. and F.C., C.S. and M.A., and F.C. and M.A.) according to the criteria in Table 1. Any disagreement was resolved by consultation with an additional reviewer (S.O.).

### 2.3. Data Extraction 

Data were extracted independently by pairs of reviewers (C.S. and F.C., F.C. and M.A., and C.S. and M.A.) and checked for accuracy by S.O. The following details were extracted from the included studies: study design, sample size, participant characteristics, experimental and comparison interventions, intervention challenge parameters (initial challenge, progression of challenge), intervention amount parameters (session duration, work duration if available, frequency per week, and program length), measurement timepoints, and study findings for measures of walking speed and/or walking endurance. Two authors (S.O. and G.A.) rated the “challenge” of each experimental and comparison intervention as “low”, “moderate”, or “high”; the criteria and supporting literature used to rate challenge are provided in Table 2.

**Table 2:** Rating of intervention “challenge” determined from training characteristics.

Challenge Rating	% Baseline Walking Speed	RPE	% HRR	% HRmax	% VO_2_ Peak	% 1-RM	Comparable Physiotherapy Interventions
Low	<90% fastest OG gait speed, <135% comf OG gait speed, or 100% comf treadmill speed	RPE <12/20	<40% HRR	<64% HRmax	<61% VO_2_ peak	Resistance training < 60% 1-RM	Stationary cycle ergometer (<5 min per session), walking between parallel bars, standing balance exercises, seated quadriceps extension, or bed exercises.
Moderate	90–110% fastest OG gait speed, 135–165% comf OG gait speed, or ≈140% comf treadmill speed	RPE 12–13/20	40–60% HRR	64–76% HRmax	61–80% VO_2_ peak	Resistance training 60–70% 1-RM	Therapeutic activities at 40–60% HRR including upright stationary cycle ergometer, recumbent bike, upper limb ergometer, stepper, cross-trainer, or stairs.
High	>110% fastest OG gait speed, >165% comf OG gait speed, or fastest possible treadmill speed	RPE >13/20 or >5/10	>60% HRR	>77% HRmax	>80% VO_2_ peak	Resistance training ≥ 80% 1-RM	

Abbreviations: % 1-RM, percentage of one-repetition maximum; comf, comfortable; % HRmax, percentage of maximal HR; % HRR, percentage of heart rate reserve; OG, overground; RPE, rating of perceived exertion; % VO_2_ peak, percentage of peak oxygen uptake. References for challenge ratings: % baseline walking speed [26,27,28,29,30], RPE [26,31], % HRR [31,32,33,34], % HRmax [31], % VO_2_ peak [35,36], % 1-RM [37], comparable physiotherapy interventions [32]. Note: In cases where challenge progressed over the course of a program, the program length, progression time point, and type of progression were considered in the challenge rating. Cognitive components of the intervention were not considered in the challenge rating.

### 2.4. Completeness of Intervention Reporting Assessment

The completeness of reporting for intervention and comparison interventions was evaluated by one reviewer (E.G.) using the TIDieR-Rehab checklist [38], an extended version of the Template for Intervention Description and Replication (TIDieR) that is specific to rehabilitation interventions [39]. Complete reporting was defined as clear and unambiguous description of an item to an extent that would allow for replication, whereas incomplete reporting was defined as no description or partial description of information pertaining to the item [40].

### 2.5. Risk-of-Bias Assessment

Studies were assessed using the revised Cochrane risk-of-bias tool (RoB2) [41] by two independent reviewers (S.O. and N.T.). The RoB2 considers bias across five domains: randomization process, deviations from the intended interventions, missing outcome data, outcome measurement, and reported results. The risk of bias was classified as low, some concerns, or high for each domain and collated to provide an overall risk assessment [41].

### 2.6. Data Processing

The studies were divided into two categories: (1) TT versus other physiotherapy interventions and (2) TT versus another type of TT. If studies recorded both walking speed and walking endurance, both outcomes were included in the meta-analyses. However, if a study reported both comfortable and fast-paced walking speed, the fast-paced data were used in the meta-analysis [27].

Using the challenge ratings (Table 2), the studies were categorized according to the challenge difference between experimental and comparison groups. The categories were Low:Low (low challenge in both groups), Moderate:Moderate (moderate challenge in both groups), Moderate:Low (moderate-challenge experimental versus low-challenge comparison group), High:Moderate (high-challenge experimental versus moderate-challenge comparison group), and High:Low (high-challenge experimental versus low-challenge comparison group). For studies that compared two types of TT of the same challenge level, the intervention of interest from the published study was assigned as the experimental intervention in the meta-analysis.

The intervention amount (in minutes) was calculated for each experimental and comparison group with the following formula: session duration × frequency per week × program length. Where data were available, session duration was replaced with work duration (i.e., session duration minus rest periods). Intervention amount included both the intervention of interest and additional interventions provided during the program (where data were provided). For each study, the difference in amount between the experimental and comparison groups was categorized as Lower (lower amount in the experimental versus the comparison group), Equal (equal amounts in both groups), or Higher (higher amount in the experimental versus the comparison group).

For the calculation of dosage in each experimental and comparison group, the challenge classification (Low = 1, Moderate = 2, High = 3) was multiplied by the amount (in hours). Then the dosage difference (challenge rank.hours) between the experimental and comparison groups was calculated for each study.

### 2.7. Meta-Analyses

Meta-analyses were performed in R software using package meta [42,43] with separate meta-analyses for walking speed and walking endurance outcomes. Random effects models were fitted to the data using the Sidik–Jonkman estimator. Hedges’ g statistic was used to calculate the standardized mean differences (SMDs) and 95% confidence intervals (CIs) across experimental and comparison groups; if available, this was calculated using the pre- to post-intervention mean change values and the standard deviation (SD) of the change or, alternatively, the pre- and post- intervention mean and SD values were utilized, assuming a correlation between pre and post measures of 0.5. Subgroup analyses were performed to evaluate the effect of the challenge difference and the amount difference across subgroups. Significant differences across subgroups were evaluated with the Chi-squared (Chi^2^) test, with a 5% type-I error rate. The heterogeneity of subgroups was evaluated with the I^2^ statistic. A random effects meta-regression model was applied with the dosage difference as a continuous covariate to determine the effect of dosage on outcomes. The F-test was used to evaluate the significance of the dosage difference with a 5% type-I error rate. See Supplementary File S2 for further information.

## 3. Results

### 3.1. Identification and Selection of Studies

**Figure 1:**
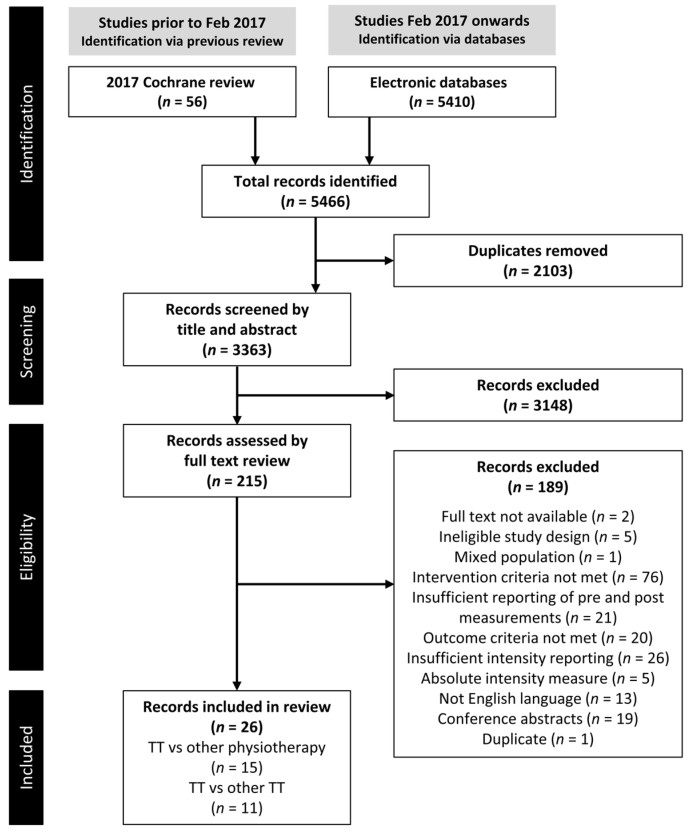
PRISMA flowchart showing the selection of studies.

The study selection is summarized in Figure 1. Following screening, 26 RCT studies were included in the review.

### 3.2. Participant Characteristics

The 26 included studies reported data for 1209 participants (36% female, mean age 59 years). Most studies enrolled participants in the chronic stage of stroke (19 studies) and only seven studies enrolled participants with subacute stroke. Baseline walking ability was assessed as part of the eligibility criteria for all but one study [44]. Approximately half of the studies required participants to walk independently for 10 meters or more. See Table 3 for the characteristics of the study participants.

### 3.3. Intervention Characteristics 

Most studies (73%) utilized TT without BWS. Of the 26 studies included, 15 studies investigated TT versus other physiotherapy interventions such as overground walking training [45,47,53,56,57], BWS overground walking training [49], conventional gait therapy [46,48,58], stepping activities [50], conventional rehabilitation (neurodevelopmental therapy) [52], stretches or range-of-motion exercises [51,55], upper and lower limb exercises [54], or relaxation [44]. The other 11 studies investigated TT versus another type of TT. Many of these studies compared TT with and without various adjuncts such as a shoe insole [61], mirror therapy [62], visual biofeedback [63], taping [64], cognitive dual tasking [66], or virtual reality [67]. Other studies compared two different types of treadmill training, such as training at high versus low intensity [36], high speed versus high incline [60], water-based TT versus standard TT [68], or TT preceded by PNF facilitation versus TT alone [65]. One study compared two different amounts of TT against a no-intervention control group [59]; as this was the only study in the review that made a comparison with an inactive control, this comparison was not considered in the meta-analysis. See Table 3 for further descriptions of the experimental and comparison interventions. 

### 3.4. Intervention Challenge 

The challenge rating for experimental and comparison groups is provided in Table 3 and the information used to classify the challenge is provided in Table 4. For the 15 studies comparing TT versus other physiotherapy, 5 studies compared 2 low-challenge interventions [49,52,53,56,57], 1 study compared 2 moderate-challenge interventions [47], 4 studies compared a moderate-challenge TT intervention against a low-challenge physiotherapy intervention [44,46,51,54], and 5 studies compared a high-challenge TT intervention against a low-challenge physiotherapy intervention [45,48,50,55,58]. For the 11 studies comparing 2 different types of TT, the majority (*n* = 9) matched the challenge level in the experimental and comparison groups; 5 studies compared 2 low-challenge TT interventions [62,63,66,67,68], 4 studies compared 2 moderate-challenge TT interventions [59,61,64,65], 1 compared high-challenge TT versus moderate-challenge TT [60], and 1 compared high-challenge TT versus low-challenge TT [36]. Subgroups of studies investigating subacute or chronic stroke investigated similar proportions of high, moderate, and low challenge interventions.

### 3.5. Intervention Amount

The intervention amount is summarized in Table 3, and further information is provided in Supplementary Table S1. Most studies matched the amount of training in the experimental and comparison groups. Only four studies compared different amounts of training; one study aimed to compare different amounts of TT training (4 months versus 2 months) [59], while the other three studies aimed to compare different types of training but did not match the amount of training, reporting either higher [52] or lower [50,58] amounts of training in the experimental TT group.

### 3.6. Walking Measurements 

The majority of studies (*n* = 16) measured both walking endurance with the 6 MWT and walking speed with the 10 mWT [36,44,45,46,47,49,51,54,56,59,60,61,62,64,65,67]. Three studies measured walking endurance only [50,55,57] and seven studies measured walking speed only [48,52,53,58,63,66,68]. Four studies measured both the comfortable and the fast-paced 10 mWT [45,46,47,59]. Measurement time points can be seen in Table 3. Of the 26 studies, only 10 measured walking outcomes at follow-up time points ranging from 4 weeks to 12 months post intervention.

### 3.7. Quality of Intervention Reporting

According to the TIDieR-Rehab checklist, the overall completeness of the intervention description was 68%, with comparable completeness in studies comparing TT versus other physiotherapy (71%) and TT versus TT (66%). Challenge and progression were better reported for TT interventions (90% and 70%, respectively) than comparison physiotherapy interventions (56% and 25%, respectively), as many comparison interventions were not set or progressed using a subjective or objective measure. As per the inclusion criteria, all studies reported session duration, frequency, and program length for all groups. However, work duration was only specified for 59% of groups. Other poorly reported sections pertained to the expertise and training of intervention providers (29%), personalization (23%), protocol deviations (7%), adherence and fidelity (38%), and adverse events (34%).

### 3.8. Risk of Bias

**Figure 2:**
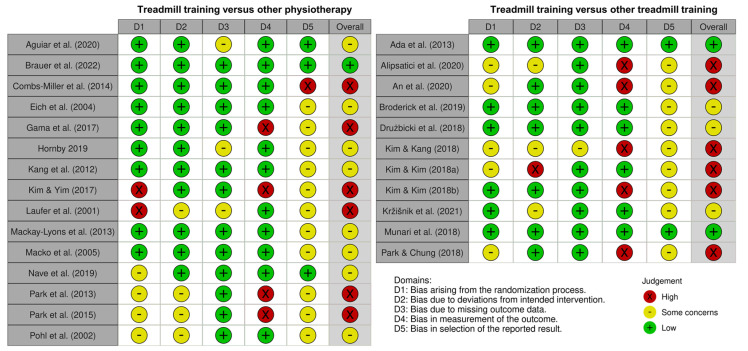
Risk of bias assessed with the revised Cochrane Risk of Bias Tool (RoB2) for studies comparing treadmill training versus other physiotherapy [44,45,46,47,48,49,50,51,52,53,54,55,56,57,58] and treadmill training versus another type of treadmill training [36,59,60,61,62,63,64,65,66,67,68].

The risk of bias varied across the studies (see Figure 2). Only three studies were deemed to have an overall low risk of bias, and therefore, the certainty of the evidence was deemed low to moderate for the categories of TT versus other physiotherapy and TT versus TT. Most studies had some bias concerns in Domain 5 due to a failure to report a pre-specified data analysis plan.

### 3.9. Walking Endurance Outcomes: Effects of Challenge, Amount, and Dosage

#### 3.9.1. Studies Comparing Treadmill Training with Other Physiotherapy

**Figure 3:**
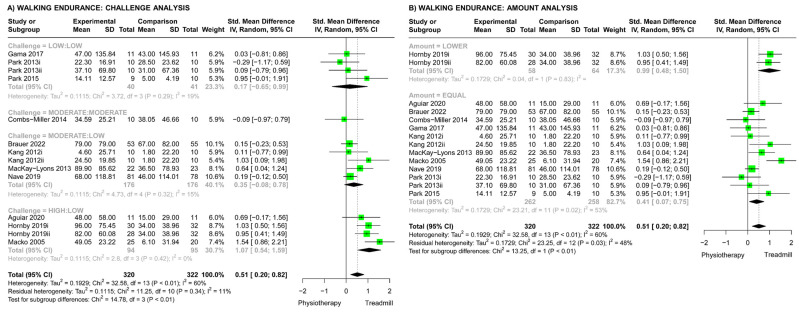
Meta-analyses for effect of treadmill training (TT) vs. physiotherapy (PT) on walking endurance. (**A**) Analysis comparing different challenge subgroups (Low:Low, low challenge in both groups [49,56,57]; Moderate:Moderate, moderate challenge in both groups [47]; Moderate:Low, moderate-challenge TT vs. low-challenge PT [44,46,51,54]; High:Low, high-challenge TT vs. low-challenge PT [45,50,55]). (**B**) Analysis comparing different amount subgroups (lower amount of TT versus PT [50]; equal amounts in both groups [44,45,46,47,49,51,54,55,56,57]).

**Figure 4:**
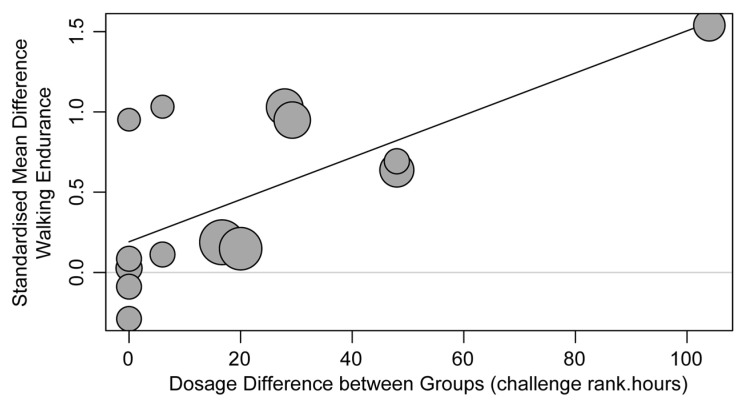
Visualization of meta-regression analysis for effects of dosage difference (between TT and other physiotherapy) on walking endurance.

For studies comparing TT versus other physiotherapy, the overall meta-analysis showed that TT resulted in significantly greater improvements in walking endurance compared with other physiotherapy (SMD 0.51, 95% CI (0.20, 0.82), Figure 3). The analysis of challenge subgroups (Figure 3A) showed a significant difference across challenge subgroups (Chi^2^ test, *p* < 0.01); this can be seen as a trend towards greater effects as the difference in challenge between TT and physiotherapy increased. In addition, there was a significant effect of high-challenge TT versus low-challenge physiotherapy on walking endurance (High:Low subgroup SMD 1.07, 95% CI (0.54, 1.59)). The analysis of amount subgroups (Figure 3B) revealed a significant difference across amount subgroups (Chi^2^ test, *p* < 0.01), favoring the subgroup with a lower amount of TT than physiotherapy. However, the heterogeneity of subgroups was high (I^2^ 48%) and only one study (two comparisons) had a lower amount in the TT group, whereas all other studies demonstrated equal amounts in both groups. The meta-regression showed a significant effect of dosage; as dosage differences increased between the TT and comparison physiotherapy groups, the effects on walking endurance increased (increase per challenge rank.hour = 0.013, 95% CI (0.005, 0.021), *p* < 0.01). This can be visualized in Figure 4.

#### 3.9.2. Studies Comparing Different Types of Treadmill Training

**Figure 5:**
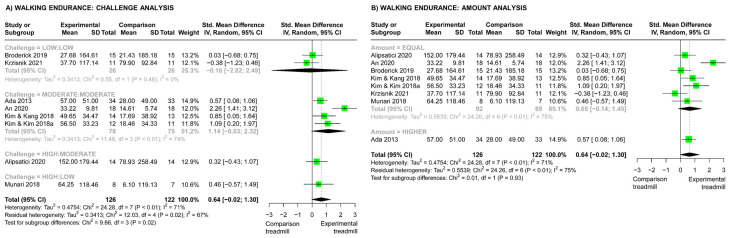
Meta-analyses for the effect of treadmill training (TT) vs. other TT interventions on walking endurance. (**A**) Analysis comparing different challenge subgroups (Low:Low, low challenge in both groups [62,67]; Moderate:Moderate, moderate challenge in both groups [59,61,64,65]; High:Moderate, high-challenge experimental TT vs. moderate-challenge comparison TT [60]; High:Low, high-challenge experimental TT vs. low-challenge comparison TT [36]). (**B**) Analysis comparing different amount subgroups (equal amounts of TT in both groups [36,60,61,62,64,65,67]; higher amount of TT in experimental group [59]).

For studies comparing two different types of TT, there was no significant difference between experimental and comparison interventions for walking endurance outcomes (SMD 0.64, 95% CI (−0.02, 1.30)). For the analysis of the challenge subgroups, there were significant differences across different challenge subgroups (Figure 5A) for walking endurance outcomes (Chi^2^ test, *p* = 0.02); this can be observed as larger effects in the studies that matched two moderate-challenge interventions compared with studies that matched two low-challenge interventions. There were no significant differences in walking endurance outcomes in the two studies by Alipsatici et al. (2020) [60] and Munari et al. (2018) [36], which compared two TT interventions of different challenge levels. For the analysis of different amount subgroups, only one study had a higher amount of TT in the experimental group [59] and the other seven studies had equal amounts in both groups. There was no significant difference across amount subgroups (Chi^2^ test, *p* = 0.93, Figure 5B). The meta-regression showed no significant effect of dosage differences on walking endurance outcomes (*p* = 0.68).

### 3.10. Walking Speed Outcomes: Effects of Challenge, Amount, and Dosage

#### 3.10.1. Studies Comparing Treadmill Training with Other Physiotherapy

**Figure 6:**
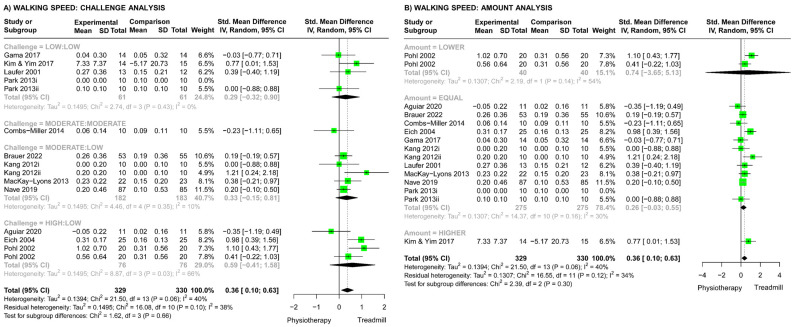
Meta-analyses for the effect of treadmill training (TT) vs. physiotherapy (PT) on walking speed. (**A**) Analysis comparing different challenge subgroups (Low:Low, low challenge in both groups [49,52,53,56]; Moderate: Moderate, moderate challenge both groups [47]; Moderate:Low, moderate-challenge TT vs. low-challenge PT [44,46,51,54]; High: Low, high-challenge TT vs. low-challenge PT [45,48,58]). (**B**) Analysis comparing different amount subgroups (lower amount of TT vs. PT [44,45,46,47,48,49,51,53,54,56]; equal amounts of TT and PT, higher amount of TT vs. PT [52]).

For studies comparing TT versus physiotherapy, the meta-analysis showed that TT resulted in significantly greater improvements in walking speed compared with other physiotherapy (SMD 0.36, 95% CI (0.10, 0.63), Figure 6). However, there were no statistically significant differences across challenge subgroups (Chi^2^ test, *p* = 0.66, Figure 6A) or amount subgroups (Chi^2^ test, *p* = 0.30, Figure 6B), and the meta-regression showed no significant effect of dosage (*p* = 0.42).

#### 3.10.2. Studies Comparing Different Types of Treadmill Training

**Figure 7:**
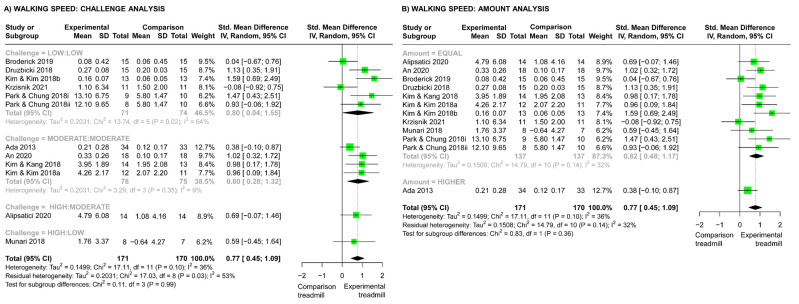
Meta-analyses for the effect of treadmill training (TT) vs. other TT interventions on walking speed. (**A**) Analysis comparing different challenge subgroups (Low:Low, low challenge in both groups [62,63,66,67,68]; Moderate:Moderate, moderate challenge in both groups [59,61,64,65]; High:Moderate, high-challenge experimental TT vs. moderate-challenge comparison TT [60]; High:Low, high-challenge experimental TT vs. low-challenge comparison TT [36]). (**B**) Analysis comparing different amount subgroups (equal amounts of TT in both groups [36,60,61,62,63,64,65,66,67,68]; higher amount of TT in experimental group [59]).

For studies comparing two different types of TT, there was a significantly larger effect of the experimental TT interventions on walking speed compared with the comparison TT interventions (SMD 0.77, 95% CI (0.45, 1.09), Figure 7). There were no significant differences across challenge subgroups (Chi^2^ test, *p* = 0.99, Figure 7A) or amount subgroups (Chi^2^ test, *p* = 0.36, Figure 7B) for walking speed outcomes, and the meta-regression showed no significant effect of dosage on walking speed (*p* = 0.45).

## 4. Discussion

This is the first systematic review to meta-analyze the effect of challenge and dosage (challenge × amount) parameters on the efficacy of TT and comparison interventions following stroke. In addition, this review presents a novel method for quantifying intervention dosage for the purpose of meta-analysis.

### 4.1. Walking Endurance Outcomes: Effects of Challenge, Amount, and Dosage

#### 4.1.1. Studies Comparing Treadmill Training with Other Physiotherapy

When comparing TT with other physiotherapy interventions, there was a significant effect of challenge on walking endurance outcomes (*p* < 0.01). With greater differences in challenge between the TT and comparison physiotherapy interventions, there were greater improvements in walking endurance. There was low to moderate certainty in this finding based on the risk-of-bias assessment. In addition, the meta-analysis of three studies with moderate risk of bias [45,50,55] that compared high-challenge TT with low-challenge physiotherapy showed a significant effect on walking endurance in favor of the high-challenge TT (SMD 1.07, 95% CI (0.54, 1.59)) (Figure 3A). Thus, these findings indicate that the challenge level of TT is an important consideration when aiming to improve walking endurance after stroke.

Studies that compared TT versus other physiotherapy largely matched the amount of training in both groups (Figure 3B). This limited the ability to determine the effect of training amount on walking endurance outcomes. The subgroup analysis showed a significant difference across amount subgroups but unexpectedly favored lower amounts of TT than physiotherapy for improving walking endurance (Figure 3B). This finding is in contrast with other stroke literature, which favors higher amounts of training [12]. The finding should be viewed with caution due to high subgroup heterogeneity (I^2^ 48%) and the inclusion of only one study that compared different amounts of training. This study, by Hornby et al. (2019) [50], had only small differences in the training amount between the two experimental TT interventions and the comparison physiotherapy intervention (TT of 891 min and 918 min versus physiotherapy of 999 min over 8 weeks) and used a high-challenge TT intervention, which likely influenced the walking endurance outcomes. These factors call into question the validity of the amount subgroup analysis. Importantly, this highlights the limitations of analyzing intervention amount without considering challenge, which has been common in meta-analyses of rehabilitation interventions [12,13,14,16]. Analyzing the effect of the amount of rehabilitation without considering challenge may provide misleading findings, and this should be considered in future research.

To address the limitations in current methods of meta-analysis of rehabilitation interventions, we explored intervention dosage, which considers both challenge and amount together. The dosage findings mimicked the findings for training challenge. That is, larger differences in training dosage between TT and comparison physiotherapy groups resulted in significantly greater improvements in walking endurance (Figure 4). Thus, a higher dosage of TT resulted in larger improvements in walking endurance for people with stroke. There was low to moderate certainty in this finding based on the risk-of-bias assessment. The investigation of dosage as a construct is relatively new to stroke literature. Amanzonwe et al. (2023) investigated how dosage influences the efficacy of aerobic training and resistance exercise after stroke [20]. Their subgroup analysis categorized studies (*n* = 11) according to the experimental intervention parameters as high dosage (amount ≥ 120 min/week and challenge ≥ 60% HRR or ≥14 RPE) or low dosage. The subgroup of studies with higher-dosage experimental interventions had larger effects on walking endurance than their comparison interventions [20]. However, the dosage of the comparison intervention was not considered in the analysis [20], nor were comparisons between the subgroups clearly reported. Furthermore, the classification of high dosage, which required dichotomization based on a threshold for both amount and challenge level, may be a limiting factor, as lower amounts of high-challenge training could be misclassified. A mathematical calculation of dosage, as used in our study (challenge rating × amount), is a potential solution to this problem. Miller et al. (2014) [69] used a similar calculation (% HRR × amount) to calculate cumulative dosage in intensity minutes in a weight-management exercise program. Although our findings suggest that dosage calculations may offer advantages over calculating challenge or amount in isolation, further research is needed to establish the best method for calculating dosage for meta-analyses. In addition, future research should investigate dosage in a group of studies that manipulates the amount and challenge of training to determine whether there is a positive relationship with walking endurance outcomes. Importantly, an increased focus on dosage in stroke rehabilitation research and exploration of the interaction between challenge and amount may highlight new opportunities to optimize rehabilitation. Given that increased challenge could be provided within current rehabilitation services without necessitating greater therapist time, this may provide avenues for improving rehabilitation outcomes without placing greater demands on services.

The mechanism for improved walking endurance may be improved cardiorespiratory capacity, as seen following other aerobic training interventions after stroke [70,71]. Bang et al. (2016) found that high-challenge versus low-challenge cycle ergometry resulted in greater improvements in both respiratory function (forced vital capacity) and walking endurance [72]. Lee et al. (2008) found that high-challenge versus low-challenge cycle ergometry and resistance training resulted in greater improvements in cardiorespiratory function (peak oxygen uptake) and walking endurance [73]. Of the six studies in this review that compared a high-challenge TT intervention with a low-challenge comparison, five [36,45,48,50,55] set the challenge level using an objective cardiorespiratory measure (%HRR, HR at %VO_2_ peak). These measures may have been chosen to specifically drive the cardiorespiratory response to TT.

#### 4.1.2. Studies Comparing Different Types of Treadmill Training

When comparing two different types of TT, none of the challenge subgroups showed a significant effect on walking endurance (Figure 5A). There was a significant difference across challenge subgroups (*p* = 0.02), but there was no relationship between higher challenge levels and larger effects on walking endurance. Instead, the largest differences in effect were seen in studies that matched two moderate-challenge TT interventions (Moderate:Moderate SMD 1.14, 95% CI (−0.03, 2.32)) and the smallest differences when two low-challenge TT interventions were compared (Low:Low SMD −0.16, 95% CI (−2.82, 2.49)) (Figure 5A). To explore this result, we inspected the four studies that matched two moderate-challenge interventions. Aside from one study that used a higher amount of training in the experimental TT group (4 months versus 2 months) [59], which likely explained the difference in effect, the other three studies investigated TT combined with another therapeutic adjunct (an insole applied to the affected foot [61], PNF taping [64], and PNF practice before TT [65]) compared with TT without the adjunct. As other measures of challenge (Borg RPE, relative walking speed) were kept constant between groups, these adjuncts did not alter the challenge rating (Table 2). This suggests that something other than challenge was responsible for the greater improvements in walking endurance in the experimental TT group that included these adjuncts. In this case, the mechanism for improvements in walking endurance could have been through improved biomechanics or neuromuscular control associated with the adjuncts, which subsequently increased walking efficiency [74,75].

There was a lack of studies investigating two different amounts of TT, and this limited the amount subgroup analysis. For comparisons of TT versus another type of TT, there was no significant difference across amount subgroups for effects on walking endurance nor a relationship between larger dosages and walking endurance outcomes. This aligns with the notion that something other than dosage parameters was responsible for differences in walking endurance outcomes between groups that investigated different types of TT.

### 4.2. Walking Speed Outcomes: Effects of Challenge, Amount, and Dosage

For both categories of studies (TT versus other physiotherapy and TT versus TT), training challenge, amount, and dosage did not significantly influence the effects on walking speed. Improvements in walking endurance but not walking speed provides further support for the underlying mechanism of TT being improved cardiorespiratory function. Meta-analyses by Stoller et al. (2012) found that aerobic training interventions following stroke, such as TT, leg cycling, and circuit training, improved cardiorespiratory function and walking endurance but not walking speed [71]. That said, our overall meta-analysis results showed that TT improved walking speed compared with other physiotherapy methods (SMD 0.36, 95% CI (0.10, 0.63), Figure 6) and other TT interventions (SMD 0.77, 95% CI (0.45, 1.09), Figure 7). Although most studies matched the amount of training in minutes, limiting our analysis of different amounts, it is possible that the use of other metrics of amount, such as the number of steps completed [76,77], may reveal the efficacy of different amounts of training [11]. It is also probable that intervention features other than amount and challenge play a role in improving walking speed. Such features could include the aforementioned adjuncts that may have altered biomechanics; TT with an insole applied to the affected foot [61] and TT with PNF taping [64] resulted in greater improvements in walking speed compared with standard TT (Figure 7). Cognitive features of the TT intervention also influenced the efficacy on walking speed. Druzbicki et al. (2018) combined TT with visual biofeedback [63] and Kim and Kim (2018b) combined TT with cognitive dual task training [66], and both resulted in greater improvements in walking speed compared with TT alone (Figure 7). This reflects how challenge can manifest in a variety of ways and should be considered in the interpretation of outcomes. For example, research in people with stroke indicates that both the physical and cognitive load of a task and how these loads interact with one another are important to neuroplastic change, task performance, and engagement in rehabilitation [78,79,80,81], which may influence outcomes [81]. However, due to limited conceptualization of challenge and effort level in the stroke rehabilitation TT literature, our challenge-rating system (Table 2) solely focused on physical load. To enable researchers to better consider the various aspects of challenge, future research should develop a classification system that goes beyond just one dimension of physical challenge.

### 4.3. Limitations

This systematic review and meta-analysis used a novel approach to classify and analyze key training parameters of TT, including challenge, amount, and dosage. However, limited conceptualization, measurement, and reporting of these parameters within the stroke rehabilitation TT literature raised several concerns during the review process.

Potentially relevant studies (*n* = 26) were excluded due to insufficient description of intervention challenge parameters. Further studies (*n* = 5) that used absolute walking speed to set the challenge level rather than relative walking speed, which accounts for the individual’s neurological impairment, were excluded. These factors narrowed the pool of studies that could be included in the meta-analysis.

For the studies that did provide sufficient description of challenge and were included, we endeavored to develop a rating system to classify challenge. However, our classification system focused on physical challenge and predominantly drew on HR measures. This classification may not reflect all the relevant aspects of challenge and may be problematic due to common confounding factors, such as the effect of beta-blockers [82] in the stroke population.

Although the included studies provided sufficient description to enable meta-analysis, reporting was not always complete. This may have limited the accuracy of our challenge, amount, and dosage data. In many cases, the challenge level of comparison physiotherapy interventions was not set according to any subjective or objective measures, and therefore the challenge rating had to be based on intervention descriptions (Table 2). This may have introduced errors in our challenge ratings. Our amount calculations also had potential for error. Several papers did not differentiate the amount of the key TT intervention and additional therapy activities, and 41% of studies did not report the work duration (active amount of intervention completed). Furthermore, the limited articulation of both challenge and amount impacted our dosage calculations, where challenge was rated according to the key TT intervention, but the amount had to be calculated based on the duration of the key intervention and additional therapy activities. This may have caused an over- or underestimation of the intervention dosage and highlights a broader need for improved measurement and reporting of challenge and amount in stroke rehabilitation research. Poor reporting also limits the ability to replicate interventions in clinical practice. Therefore, it is recommended that future research report the initial setting and progression of challenge for both the intervention and the comparison group using validated measures that can be replicated in practice. This will also improve accuracy when meta-analyzing challenge and dosage parameters in future research.

This study did not evaluate the effect of challenge, amount, and dosage according to different stroke types, stage of stroke, or disability level, and did not look at effects on other measures of walking impairment, such as gait pattern. We did not analyze outcomes at follow-up time points, as only 10 studies reported follow-up time points and the duration of time between the completion of the intervention and follow-up varied considerably across the studies.

### 4.4. Clinical Implications

The findings of this review suggest that clinicians should go beyond considering the amount of rehabilitation delivered during TT and also consider the challenge level and overall dosage. Based on the findings, it is recommended that, when aiming to increase walking endurance in people with stroke, physiotherapists should consider implementing TT interventions at a higher challenge level. This recommendation fits with clinical guidelines, which encourage moderate- to high-challenge walking rehabilitation to improve locomotor function in people with chronic stroke [83]. Clinicians considering implementing high-challenge TT should consider any contraindications or cautions to exercise and physiological monitoring for safety [83], particularly in the early stages after stroke [84].

Although the limitations in reporting intervention parameters present a barrier to clinical replication, our review highlights some of the key dosage parameters clinicians should draw upon when replicating TT in clinical practice—namely, session duration, frequency, program length, initial challenge level, and progression of challenge. We encourage clinicians to set and progress the challenge level using standardized outcome measures such as %HRR or Borg RPE. Furthermore, clinicians should consider how interventions can be personalized to the individual with stroke.

## 5. Conclusions

This systematic review and meta-analysis used a novel approach to classify and analyze key drivers of TT efficacy in people with stroke, including challenge, amount, and dosage. The analysis demonstrated that some of the efficacy of TT on walking endurance after stroke can be explained by the challenge level during training. Specifically, for improving walking endurance, there is low to moderate certainty that TT is more effective than other physiotherapy interventions when it is delivered at a much higher challenge level. In addition, larger differences in dosage between TT and comparison physiotherapy interventions were associated with greater effects on walking endurance; there is low to moderate certainty in this finding. However, when two different types of TT were compared, greater levels of challenge and dosage did not influence walking endurance, possibly due to the effects of therapeutic adjuncts that did not alter the challenge rating. Furthermore, walking speed outcomes were not influenced by challenge or dosage parameters. This result may suggest that intervention features other than challenge are required to increase walking speed or may reflect limitations in the approach used to quantify challenge. The amount analysis was limited by a lack of studies that compared interventions of varying amounts. Future research should prioritize investigating the effects of dosage to better understand and optimize rehabilitation interventions and stroke outcomes. This will first require research to improve the conceptualization, measurement, and reporting of challenge and dosage parameters. The findings have implications for clinical practice and suggest that physiotherapists should consider implementing treadmill training at a higher challenge level for enhanced walking endurance after stroke.

## Figures and Tables

**Table 1:** Eligibility criteria.

	Inclusion	Exclusion
Participants	Aged ≥ 18 years with stroke diagnosis and lower limb motor deficit resulting in slow gait speed or abnormal gait pattern.	
Experimental intervention	TT with or without BWS, either alone or in combination with another intervention.	Robotic or mechanically assisted TT. Backwards TT only. Non-invasive brain stimulation. Single-session intervention.
Comparison intervention	Either another physiotherapy intervention, another type of TT (±BWS) alone or in combination with another intervention, or no intervention.	As per exclusion criteria for experimental intervention.
Outcomes	Pre- and post-intervention measurements of either unassisted walking speed (with 6 m or 10 m walk test (10 mWT)) and/or unassisted walking endurance (with 6 min walk test (6 MWT)).	
Data reported	Sufficient description of interventions to enable extraction of intervention amount (in minutes) and rating of challenge for both experimental and comparison groups according to Table 2.	
Study design	Randomized and quasi-randomized controlled trials.	Crossover or non-experimental studies.
Publication	Full-text peer-reviewed journal articles in English.	Conference abstracts.

**Table 3:** Study characteristics.

Author	Population Criteria	Experimental Intervention (EXP)			Comparison Intervention (COMP)			Outcome Time Points
		Description and Sample	Amount	Challenge	Description and Sample	Amount	Challenge	
**Treadmill Training Versus Other Physiotherapy Interventions**								
Aguiar et al. (2020) [45]	Chronic stroke (*n* = 22), able to walk ind ± aids for ≥10 min	Aerobic TT, *n* = 11, 3F, age 52 y (11 y), post stroke 51 m (68 m)	40 min, 3 times/w, 12 w Total 1440 min	High	Outdoor OGT, *n* = 11, 3F, age 48 y (10 y), post stroke 44 m (26 m)	40 min, 3 times/w, 12 w, total 1440 min	Low	Baseline, 12 w, 16 w
Brauer et al. (2022) [46]	Subacute stroke (*n* = 119), able to walk 10 m ind ± aids	TT + self-management education + conv GT, *n* = 60, 12F, age 62 y (11 y), post stroke 28 d (15 d)	TT: 30 min, 3 times/w, 8 w Conv GT: 30 min, 2 times/w, 8 w Total 1200 min	Mod	Conv GT, *n* = 59, 12F, age 64 y (9 y), post stroke 27 d (16 d)	30 min, 5 times/w, 8 w, total 1200 min	Low	Baseline, 8 w, 26 w
Combs-Miller et al. (2014) [47]	Chronic stroke (*n* = 20), able to walk 10 m ± aids at comf speed < 1 m/s	BWSTT, *n* = 10, 6F, age 56 y (8 y), post stroke 62 m (49 m)	30 min, 5 times/w, 2 w Total 300 min	Mod	OGT, *n* = 10, 3F, age 66 y (7 y), post stroke 60 m (52 m)	30 min, 5 times/w, 2 w Total 300 min	Mod	Baseline, 2 w, 3 m
Eich et al. (2004) [48]	Subacute stroke (*n* = 50), able to walk ≥ 12 m with intermittent help or supervision	BWSTT + conv GT, *n* = 25, 8F, age 62 y (5 y), post stroke 6.1 w (2.2 w)	TT: 30 min, 5 times/w, 6 w Conv GT: 30 min, 5 times/w, 6 w. Total 1800 min	High	Conv GT, *n* = 25, 9F, age 64 y (6 y), poststroke 6.3 w (2.5 w)	60 min, 5 times/w, 6 w Total 1800 min	Low	Baseline, 6 w, 18 w
Gama et al. (2017) [49]	Chronic stroke (*n* = 32), able to walk 10 m ± assistance	BWSTT, *n* = 14, 7F, age 59 y (8 y), post stroke 60 m (55 m)	45 min, 3 times/w, 6 w Total 810 min	Low	BWS OGT, *n* = 14, 8F, age 58 y (10 y), post stroke 54 m (42 m)	45 min, 3 times/w, 6 w Total 810 min	Low	Baseline, 7 w, 12 w
Hornby et al. (2019) [50]	Chronic stroke (*n* = 97), able to walk ind ± aids at comf gait speed < 1.0 m/s over 10 m	EXP 1: TT + OGT, *n* = 30, 13F, age 60 y (95% CI: 56–64 y), post stroke 31 m (95% CI: 19–42 m) EXP 2: Variable training on TT, OGT + stairs, *n* = 28, 5F, age 59 y (95% CI: 55–62 y), post stroke 60 m (95% CI: 14–106 m)	EXP 1: 33 min, mean 3.375 times/w, 8 w Total 891 min EXP 2: 34 min, mean 3.375 times/w, 8 w Total 918 min	EXP 1 + 2: High	Stepping activities in variable contexts, *n* = 32, 14F, age 56 y (95% CI: 52–60 y), post stroke 27 m (95% CI: 18–36 m)	37 min per session, 3–5 times/w, for 8 w Total 999 min	Low	Baseline, 8 w, 12 w
Kang et al. (2012) [51]	Chronic stroke (*n* = 32), able to walk ind for ≥15 min	EXP 1: TT + conv PT, *n* = 10, 6F, age 56 y (8 y), post stroke 14 m (4 m). EXP 2: TT + optic flow + conv PT, *n* = 10, 4F, age 56 y (7 y), post stroke 14 m (4 m)	EXP 1 + 2: 30 min, 3 times/w, 4 w Total 360 min. (session duration for conv PT 5 times/w NR)	EXP 1 + 2: Mod	Stretching, ROM exercises + conv PT, *n* = 10, 4F, age 56 y (8 y), post stroke 15 m (7 m)	30 min, 3 times/w, 4 w Total 360 min. (session duration for conv PT 5 times/w NR)	Low	Baseline, 4 w
Kim and Yim (2017) [52]	Chronic stroke (*n* = 30), able to ind walk 10 m	TT + handgrip strengthening + conv rehab (NDT), *n* = 14, 5F, age 51 y (15 y), post stroke 13 m (7 m)	TT + hand strengthening: 35 min, 3 times/w, 6 w Conv rehab: 60 min, twice daily, 5 times/w, 6 w Total 4230 min	Low	Conv rehab (NDT), *n* = 15, 5F, age 52 y (17 y), post stroke 12 m (8 m)	60 min, twice daily, 5 times/w, 6 w Total 3600 min	Low	Baseline, 6 w
Laufer et al. (2001) [53]	Subacute stroke (*n* = 25), able to walk 2 min on treadmill at ≥0.2 km/h with mod assistance	TT + conv PT, *n* = 13, 6F, age 67 y (7 y), post stroke 33 d (21 d)	4–8 min, 5 times/w, 3 w Total 90 min (session duration for conv PT 5 times/w NR)	Low	OGT + conv PT, *n* = 12, 5F, age 69 y (8 y), post stroke 36 d (17 d)	4–8 min, 5 times/w, 3 w Total 90 min (session duration for conv PT 5 times/w NR)	Low	Baseline, 3 w
MacKay-Lyons et al. (2013) [54]	Subacute stroke (*n* = 50), able to walk 5 m ± aids or stand-by assistance	BWSTT + stretching + UL/LL active exercises, *n* = 24, 9F, age 62 y (15 y), post stroke 23 d (6 d)	60 min, 5 times/w for 1st 6 w and 3 times/w for 2nd 6 w Total 2880 min	Mod	Stretching + UL/LL active exercises, *n* = 26, 12F, age 59 y (13 y), post stroke 29 d (6 d)	60 min, 5 times/w for 1st 6 w and 3 times/w for 2nd 6 w Total 2880 min	Low	Baseline, 12 w, 6 m, 12 m
Macko et al. (2005) [55]	Chronic stroke (*n* = 61), able to walk on treadmill for 3 min at >0.22 m/s	TT, *n* = 32, 10F, age 63 y (10 y)	40 min, 3 times/w, 26 w Total 3120 min	High	Supervised stretches + 5 min TT, *n* = 29, 8F, age 64 y (8 y), post stroke 39 m (59 m)	40 min, 3 times/w, 26 w Total 3120 min	Low	Baseline, 6 m
Nave et al. (2019) [44]	Subacute stroke (*n* = 200)	BWSTT + conv rehab, *n*= 105, 45F, age 69 y (12 y), post stroke 30 d (IQR: 17–39 d)	50 min, 5 times/w, 4 w Total 1000 min	Mod	Relaxation + conv rehab, *n* = 95, 36F, age 70 y (11 y), post stroke 27 d (IQR: 17–41 d)	50 min, 5 times/w, 4 w Total 1000 min	Low	Baseline, 4 w, 3 m, 6 m
Park et al. (2013) [56]	Chronic stroke (*n* = 40), able to walk ≥ 10 m without aids	TT. Analyzed in 2 groups: slow walkers (*n* = 10, 3F, age 52 y (9 y) post stroke 23 m (6 m)), and fast walkers (*n* = 10, 5F, age 53 y (7 y) post stroke 19 m (8 m))	30 min, 10 times/w (twice daily), 1 w Total 300 min	Low	OGT. Analyzed in 2 groups: slow walkers (*n* = 10, 4F, age 51 y (11 y) post stroke 17 m (9 m)) and fast walkers (*n* = 10, 3F, age 55 y (7 y) post stroke 15 m (7 m))	30 min, 10 times/w (twice daily), 1 w Total 300 min	Low	Baseline, 4 w
Park et al. (2015) [57]	Chronic stroke (*n* = 19), able to walk ≥ 10 min on treadmill	TT with rhythmic auditory stimulation + conv rehab (NDT), *n* = 9, 5F, age 52 y (13 y), post stroke 10 m (3 m)	30 min, 5 times/w, 3 w Total 450 min. (session duration for conv rehab 5 times/w NR)	Low	OGT with rhythmic auditory stimulation + conv rehab (NDT), *n* = 10, 4F, age 55 y, post stroke 13 m (4 m)	30 min, 5 times/w, 3 w Total 450 min. (session duration for conv rehab 5 times/w NR)	Low	Baseline, 3 w
Pohl et al. (2002) [58]	Subacute stroke (*n* = 60), able to walk ind 10 m in 5–60 s	EXP 1: Speed dependent TT + conv PT, *n* = 20, 4F, age 58 y (11 y), post stroke 16 w (16 w) EXP 2: Limited progressive TT + conv PT, *n* = 20, 6F, age 57 y (14 y), post stroke 17 w (21 w)	TT: 30 min, 3 times/w, 4 w Conv PT: 45 min, 2 times/w, 4 w Total 720 min	EXP 1 + 2: High	Conv GT + conv PT, *n* = 20, 7F, age 62 y (11 y), post stroke 16 w (19 w)	Conv GT: 45 min, 3 times/w, 4 w Conv PT: 45 min, 2 times/w, 4 w Total 900 min	Low	Baseline, 4 w
**Treadmill Training Versus Another Type of Treadmill Training**								
Ada et al. (2013) [59]	Chronic stroke (*n* = 102), able to walk 10 m without aids in >9 s	EXP 1: 4 m TT + OGT, *n* = 34, 10F, age 70 y (11 y), post stroke 22 m (16 m)	30 min, 3 times/w, 16 w Total 1440 min	Mod	COMP 1: 2 m TT + OGT, *n* = 34, 6F, age 64 y (12 y), post stroke 20 m (15 m) COMP 2: No intervention (not meta- analyzed), *n* = 34, 15F, age 63 y (13 y), post stroke 19 m (13 m)	COMP 1: 30 min, 3 times/w, 8 w Total 720 min COMP 2: 0 min	Mod	Baseline, 2 m, 4 m, 6 m, 12 m
Alipsatici et al. (2020) [60]	Chronic stroke (*n* = 30), able to ind walk 10 m in <0.9 m/s	TT with increased speed + conv rehab + e-stim, *n* = 14, 6F, age 45 y (12 y), post stroke NR	75 min (TT 30, conv rehab 30, e-stim 15 min), 3 times/w, 8 w Total 1800 min	High	TT with increased incline + conv rehab + e-stim, *n* = 14, 5F, age 40 y (12 y), post stroke NR	75 min (TT 30, conv rehab 30, e-stim 15 min), 3 times/w, 8 w Total 1800 min	Mod	Baseline, 8 w
An et al. (2020) [61]	Chronic stroke (*n* = 36), functional ambulation category (FAC) score ≥ 3	Insole during TT + conv PT, *n* = 18, 7F, age 55 y (11 y), post stroke 10 m (2 m)	30 min, 5 times/w, 4 w Total 600 min (session duration for conv PT NR)	Mod	TT + conv PT, *n* = 18, 6F, age 55 y (9 y), post stroke 10 m (2 m)	30 min, 5 times/w, 4 w Total 600 min. (session duration for conv PT NR)	Mod	Baseline, 4 w
Broderick et al. (2019) [62]	Chronic stroke (*n* = 30), ambulatory ± aids	TT + mirror therapy, *n* = 15, 5F, age 61 y (10 y), post stroke 75 m (88 m)	30 min, 3 times/w, 4 w Total 360 min	Low	TT + placebo mirror therapy, *n* = 15, 5F, age 67 y (19 y), post stroke 34 m (31 m)	30 min, 3 times/w, 4 w Total 360 min	Low	Baseline, 6 w, 3 m
Drużbicki, et al. (2018) [63]	Chronic stroke (*n* = 30), able to walk ind	BWSTT with visual biofeedback + conv rehab, *n* = 15, 5F, age 62 y (10 y), post stroke 9 d (6–23 d)	TT: 30 min, 5 times/w, 3 w Conv rehab: 120 min, 5 times/w, 3 w + 45 min, once per w, 3 w Total 2385 min	Low	BWSTT without visual biofeedback + conv rehab, *n* = 15, 7F, age 62 y (11 y), post stroke 8 d (5–19 d)	TT: 30 min, 5 times/w, 3 w Conv rehab: 120 min, 5 times/w, 3 w + 45 min, once per week, 3 w Total 2385 min	Low	Baseline, 3 w
Kim and Kang (2018) [64]	Chronic stroke (*n* = 27), able to walk 10 m ind and walking speed >0.5 m/s	TT with PNF taping, *n* = 14, 6F, age 51 y (3 y), post stroke 20 m (4 m)	30 min, 5 times/w, 6 w Total 900 min	Mod	TT with placebo taping, *n* = 13, 6F, age 52 y (3 y), post stroke 21 m (3 m)	30 min, 5 times/w, 6 w Total 900 min	Mod	Baseline, 6 w
Kim and Kim (2018) [65]	Chronic stroke (*n* = 23), able to walk ind 10 m	TT + PNF, *n* = 12, 5F, age 61 y (3 y), post stroke 20 m (4 m)	40 min, (TT of 15 min), 5 times/w, 6 w Total 1200 min	Mod	TT, *n* = 11, 4F, age 61 y (3 y), post stroke 19 m (4 m)	40 min (TT of 30 min), 5 times/w, 6 w Total 1200 min	Mod	Baseline, 6 w
Kim and Kim (2018) [66]	Chronic stroke (*n* = 26), able to walk ind unaided + on treadmill > 30 min	TT with cognitive dual-task gait training, *n* = 13, 5F, age 53 y (10 y), post stroke 13 m (4 m)	30 min, 5 times/w, 4 w Total 600 min	Low	TT with no cognitive tasks, *n* = 13, 6F, age 56 y (11 y), post stroke 11 m (4 m)	30 min, 5 times/w, 4 w Total 600 min	Low	Baseline, 4 w
Kržišnik et al. (2021) [67]	Subacute stroke (*n* = 22), able to walk ind or with supervision ± aids	TT with virtual reality + conv rehab, *n* = 11, 4F, age 60 y (8 y), post stroke 5 m (2 m)	TT: mean 15.5 min, 5 times/w, 4 w Conv rehab: 90 min, 5 times/w, 4 w Total 2110 min	Low	TT + conv rehab, *n* = 11, 3F, age 55 y (6 y), post stroke 5 m (2 m)	TT: mean 15.5 min, 5 times/w, 4 w Conv rehab: 90 min, 5 times/w, 4 w Total 2110 min	Low	Baseline, 4 w
Munari et al. (2018) [36]	Chronic stroke (*n* = 16), able to walk on treadmill ≥ 0.3 km/h for 3 min	High-intensity TT, *n* = 8, 1F, age 61 y (6 y), post stroke 5 y (3 y)	55 min, 3 times/w, 12 w Total 1980 min	High	Low-intensity TT, *n* = 7, 0F, age 62 y (11 y), post stroke 6 y (4 y)	55 min, 3 times/w, 12 w Total 1980 min	Low	Baseline, 3 m
Park and Chung et al. (2018) [68]	Chronic stroke (*n* = 27), able to walk 10 min ± aids	EXP 1: Aquatic TT + conv PT, *n* = 9, 3F, age 63 y (13 y), post stroke 7 m (1 m) EXP 2: Anti-gravity TT + conv rehab, *n* = 8, 4F, age 66 y (10 y), post stroke 6.75 m (1 m)	TT: 30 min, 3 times/w, 4 w Conv PT: 30 min, 5 times/w, 4 w Total 960 min	Low	TT + conv PT, *n* = 10, 5F, age 67 y (8 y), post stroke 8 m (1 m)	TT: 30, 3 times/w, 4 w Conv PT: 30 min, 5 times/w, 4 w Total 960 min	Low	Baseline, 4 w

Abbreviations: age, mean age (standard deviation); BWSTT, bodyweight-supported treadmill training; CI, confidence interval; comf, comfortable; conv GT, conventional gait training; conv, conventional; d, days; e-stim, electrical stimulation; F, number of females; ind, independently; IQR, interquartile range; km/h, kilometers/hour; LL, lower limb; m, months; m/s, meters/second; min, minutes; mod, moderate; *n*, number; NR, not reported; NDT, neurodevelopmental therapy; OGT, overground training; PNF, proprioceptive neuromuscular facilitation; PT, physiotherapy; rehab, rehabilitation; ROM, range of motion; sec, seconds; times/w, times/week; TT, treadmill training; UL, upper limb; w, weeks; y, years.

**Table 4:** Intervention challenge.

Author	Experimental Intervention			Comparison Intervention		
	Initial Challenge Level	Progression	Overall Challenge	Initial Challenge Level	Progression	Overall Challenge
**Treadmill Training Versus Other Physiotherapy Interventions**						
Aguiar et al. (2020) [45]	Obj: 60–80% HRR	Individually tailored to ability, RPE, HR, and BP	High	Obj: <40% HRR	NR	Low
Brauer et al. (2022) [46]	Obj: 40% HRR or Subj: Borg RPE 11–14	↑ Challenge weekly until 60% HRR reached	Mod	Described as usual gait training with supervision	Individually tailored	Low
Combs-Miller et al. (2014) [47]	Subj: Borg RPE 11–14, 30% BWS	↑ Speed to target Borg RPE 11–14 ↓ Verbal/manual cues each session If speed > 2.0 mph, <3 breaks were required, and gait quality was maintained without assistance, ↓ BWS from 30% to 15% to 0%	Mod	Subj: Borg RPE 11–14	↑ Speed to keep Borg RPE 11–14 ↓ Verbal/manual cues at each session	Mod
Eich et al. (2004) [48]	Obj: 60% HRR	↑ Speed or incline to achieve target HR	High	Described as Bobath-oriented gait rehab	NR	Low
Gama et al. (2017) [49]	Subj: comf treadmill speed (patient judgement)	↓ BWS, ↑ speed, and/or ↓ hand support	Low	Subj: comf OG gait speed (patient judgement)	↓ BWS, ↑ speed, and/or ↓ hand support	Low
Hornby et al. (2019) [50]	EXP 1 + 2: Obj: 70–80% HRR	EXP 1: ↑ speed to achieve target %HRR EXP 2: ↑ speed to achieve target %HRR, ↓ handrail use, and ↑ task variation	EXP 1 + 2: High	Obj: 30–40% HRR	Stepping activities in variable contexts but maintaining 30–40% HRR	Low
Kang et al. (2012) [51]	EXP 1 + 2: Obj: Stable OG gait speed (from 10 mWT)	EXP 1 + 2: ↑ speed 0.1 km/h each time individual could walk 20 s with stable gait (+ optic flow for EXP 1)	EXP 1 + 2: Mod	Described as range-of-motion exercises	NR	Low
Kim & Yim (2017) [52]	Subj: comf treadmill speed (patient judgement)	↑ Speed by 0.1 km/h each round	Low	Described as facilitation in supine, sitting, standing + walking	NR	Low
Laufer et al. (2001) [53]	Subj: comf treadmill speed (patient judgement)	↓ Physical assistance	Low	Subj: comf OG gait speed (patient judgement)	NR	Low
MacKay-Lyons et al. (2013) [54]	Obj: HR achieved at 40–50% VO_2_ peak, 70–90% comf OG gait speed with 20–40% BWS	↑ To HR equivalent to 60–75% baseline VO_2_ peak by weeks 4–5, ↑ speed + incline, ↓ BWS + assistance	Mod	Subj: comf OG gait speed (patient judgement)	NR	Low
Macko et al. (2005) [55]	Obj: 40–50% HRR	↑ %HRR by 5% every 2 weeks to target 60–70% HRR	High	Subj: stretching (therapist judgement) Obj: 5 min TT at 30–40% HRR	NR	Low
Nave et al. (2019) [44]	Obj: 180 minus age (equating to > 64% HRmax)	NR	Mod	Described as contraction and relaxation of muscle groups (face/arms/trunk)	NR	Low
Park et al. (2013) [56]	Subj: comf treadmill speed (patient judgement)	Maintained comf treadmill speed	Low	Described as walking on 30 m track with therapist supervising behind	NR	Low
Park et al. (2015) [57]	Obj: 90% comf OG gait speed (from 10 mWT)	↑ Speed by 10% each week	Low	Obj: 90% comf OG gait speed (from 10 mWT)	↑ Speed by 10% each week	Low
Pohl et al. (2002) [58]	EXP 1: Subj: fastest safe treadmill speed (therapist judgement) EXP 2: Obj: fastest OG gait speed (from 10 mWT)	EXP 1: after 10 s successful walking, ↑ speed by 10% or ↓ if not successful EXP 2: ↑ speed up to 5% weekly (up to maximum 20%)	EXP 1 + 2: High	Described as gait therapy based on PNF + Bobath principles	NR	Low
**Treadmill Training Versus Another Type of Treadmill Training**						
Ada et al. (2013) [59]	Subj: comf treadmill speed (patient judgement) + instructions to walk slowly to ↑ step length	↑ Speed until step length compromised, ↑ incline, concurrent cognitive task OGT ↑ from 20% to 50% of session with ↑ workload	Mod	Subj: comf treadmill speed (patient judgement) + instructions to walk slowly to ↑ step length	↑ Speed until step length compromised, ↑ incline, add cognitive task. OGT ↑ from 20% to 50% of session with ↑ workload	Mod
Alipsatici et al. (2020) [60]	Obj: 80% fastest OG gait speed (from 10 mWT)	↑ Speed by 10% each week	High	Obj: 80% fastest OG gait speed (from 10 mWT) with 3% incline	↑ Incline 1–1.5% each week as able with Borg RPE 11–14	Mod
An et al. (2020) [61]	Obj: fastest OG gait speed (from 10 mWT)	↑ Speed by 10% week 1 + 20% week 2	Mod	Obj: fastest OG gait speed (from 10 mWT)	↑ Speed by 10% week 1 + 20% week 2	Mod
Broderick et al. (2019) [62]	Obj: comf treadmill speed (guided by OG 10 mWT) with mirror	NR	Low	Obj: comf treadmill speed (guided by OG 10 mWT) without mirror	NR	Low
Drużbicki, et al. (2018) [63]	Obj: comf OG gait speed (from 3D gait analysis)	↑ Step length and speed by 5–10% each session ↓ BWS	Low	Obj: comf OG gait speed (from 3D gait analysis)	↑ Speed by 5–10% each session ↓ BWS	Low
Kim and Kang (2018) [64]	Subj:11–15 Borg RPE	NR	Mod	Subj: 11–15 Borg RPE	NR	Mod
Kim and Kim (2018a) [65]	Subj: 11–15 Borg RPE	NR	Mod	Subj: 11–15 Borg RPE	NR	Mod
Kim and Kim (2018b) [66]	Subj: comf treadmill speed (patient judgement)	If stable gait was maintained for 20 s, ↑ speed 0.1 km/h next session + different cognitive tasks each week	Low	Subj: comf treadmill speed (patient judgement)	If stable gait was maintained for 20 s, ↑ speed by 0.1 km/h next session	Low
Kržišnik et al. (2021) [67]	Subj: comf treadmill speed (patient judgement)	Maintained comf speed In weeks 3 + 4, ↑ duration, ↑ speed, ↑ VR obstacles + cognitive load	Low	Subj: comf treadmill speed (patient judgement)	Maintained comf speed Stimulated stepping over/around obstacles + narrow walking	Low
Munari et al. (2018) [36]	Obj: HR achieved at 85–95% VO_2_ peak	↑ Speed + incline to maintain HR achieved at 85–95% VO_2_ peak	High	Obj: HR achieved at 60% VO_2_ peak	NR	Low
Park and Chung et al. (2018) [68]	EXP 1 +2 Obj: timed to comf gait speed (from OG 10 mWT)	NR	Low	Obj: comf gait speed (from 10 mWT)	NR	Low

Abbreviations: 10 mWT, 10 m walk test; Borg RPE, Borg rating of perceived exertion; BWS, bodyweight support; comf, comfortable; COMP, comparison; EXP, experimental; HR, heart rate; HRmax, heart rate max; HRR, heart rate reserve; km/h, kilometers/hour; mod, moderate; mph, miles per hour; NDT, neurodevelopmental training; NR, not reported; obj, objective; OG, overground; OGT, over-ground training; PNF, proprioceptive neuromuscular facilitation; SS, self-selected; SSWS, self-selected walking speed; subj, subjective; TT, treadmill training; VO_2_, volume of oxygen consumption; VR, virtual reality; ↑ increased; ↓ decreased.

## Data Availability

Primary data were obtained from the original publications of the included articles. Codes for meta-analyses can be found in the Supplementary Material.

## Supplementary Materials

The following supporting information can be downloaded at: https://www.mdpi.com/article/10.3390/brainsci13121729/s1. Supplementary Table S1: Intervention amount; Supplementary File S1: Search strategy; Supplementary File S2: Meta-analysis.
