# Supplementary material for: Challenge Level Contributes to the Efficacy of Treadmill Interventions after Stroke: A Systematic Review and Meta-Analysis
Source: Brain Sci. 2023 Dec 18;13(12):1729. doi: 10.3390/brainsci13121729 (PMC10741427; doi:10.3390/brainsci13121729)
Supplement: Supplementary file 1 [file brainsci-13-01729-s001.zip › Supplementary File S1.pdf]

Supplementary File S1: Scopus Database Search Strategy

Adapted from: Mehrholz, J., Thomas, S., & Elsner, B. (2017). Treadmill training and body weight support for walking after stroke. Cochrane Database Syst Rev, 8, CD002840. https://doi.org/10.1002/14651858.CD002840.pub4

#1. (Stroke or poststroke or “post-stroke” or cerebrovasc* or “brain vasc*” or “cerebral vasc*” or cva* or apoplex* or SAH)
#2. (brain* or cerebr* or cerebell* or intracran* or intracerebral) W/5 (ischaemi* or ischemi* or infarct* or thrombo* or emoboli* or occlu*)
#3. (brain* or cerebr* or cerebell* or intracerebral or intracranial or subarachnoid) W/5 (haemorrhage* or hemorrhage* or haematoma* or hematoma* or bleed*)
#4. hemipleg* or hemipar* or paresis or paretic
#5. (gait or walk* or locomot*) W/2 (impair* or problem* or deficit* or compromise)
#6. #1 OR #2 OR #3 OR #4 OR #5
#7. “Body weight” or “weight bearing” or weightbear*
#8. Treadmill* or “tread mill*” or “running wheel*” or “running machine*”
#9. (walk or walking or exercise) W/5 (machine* or device*)
#10. (walking or gait or locomot* or ambulat*) W/5 (train* or “re train*” or retrain*)
#11. (Weight or bodyweight or “body weight”) W/5 (support* or suspen* or relief)
#12. (Walk or walking or ambulat* or locomot* or gait or overhead) W/5 (support*)
#13. harness*
#14. #7 OR #8 OR #9 OR #10 OR #11 OR #12 OR #13
#15. walk* or gait* or ambulat* or mobil* or locomot* or stride
#16. random* W/5 (trial* or stud*)
#17. control* W/5 (trial* or stud*)
#18. (singl* or doubl* or tripl* or trebl*) W/5 (blind* or mask*)
#19. placebo* or sham* or cross-over or “cross over” or “trial*” or RCT
#20. (clinical* W/5 trial*)
#21. (control* or treatment* or experiment* or intervention) W/5 (group* or subject* or patient*)
#22. (“quasi random*” or “pseudo random*”)
#23. (control or experiment* or conservative) W/5 (treatment or therap* or procedure or manage*)
#24. assign* or allocat*
#25. #16 OR #17 OR #18 OR #19 OR #20 OR #21 OR #22 OR #23 OR #24
#26. #6 AND #14 AND #15 AND #25
