# Supplementary material for: Challenge Level Contributes to the Efficacy of Treadmill Interventions after Stroke: A Systematic Review and Meta-Analysis
Source: Brain Sci. 2023 Dec 18;13(12):1729. doi: 10.3390/brainsci13121729 (PMC10741427; doi:10.3390/brainsci13121729)
Supplement: Supplementary file 1 [file brainsci-13-01729-s001.zip › Supplementary File S2.pdf]

Meta-analysis: Dose-response relationship in Treadmill Training

Usman Rashid

09/09/2023

Contents

1 Results **2**
1.1 Treadmill vs Other . . . 2
1.1.1 Walking Endurance . . . 2
1.1.1.1 Intensity . . . 2
1.1.1.2 Amount . . . 4
1.1.1.3 Dosage . . . 6
1.1.2 Walking Speed . . . 7
1.1.2.1 Intensity . . . 7
1.1.2.2 Amount . . . 9
1.1.2.3 Dosage . . . 11
1.2 Treadmill vs Treadmill . . . 12
1.2.1 Walking Endurance . . . 12
1.2.1.1 Intensity . . . 12
1.2.1.2 Amount . . . 14
1.2.1.3 Dosage . . . 15
1.2.2 Walking Speed . . . 16
1.2.2.1 Intensity . . . 16
1.2.2.2 Amount . . . 18
1.2.2.3 Dosage . . . 19

1 Results

1.1 Treadmill vs Other

1.1.1 Walking Endurance

1.1.1.1 Intensity

```
                      SMD            95%-CI %W(random)         Intensity
Aguiar 2020        0.6923 [-0.1731; 1.5577]        6.0          LOW:HIGH
Brauer 2022        0.1479 [-0.2299; 0.5257]       10.1      LOW:MODERATE
Combs-Miller 2014 -0.0884 [-0.9654; 0.7887]        5.9 MODERATE:MODERATE
Gama 2017          0.0273 [-0.8085; 0.8631]        6.2           LOW:LOW
Hornby 2019i       1.0296 [ 0.4975; 1.5616]        8.7          LOW:HIGH
Hornby 2019ii      0.9491 [ 0.4124; 1.4857]        8.6          LOW:HIGH
Kang 2012i         0.1116 [-0.7657; 0.9890]        5.9      LOW:MODERATE
Kang 2012ii        1.0323 [ 0.0867; 1.9778]        5.4      LOW:MODERATE
MacKay-Lyons 2013  0.6377 [ 0.0372; 1.2382]        8.1      LOW:MODERATE
Macko 2005         1.5392 [ 0.8639; 2.2145]        7.4          LOW:HIGH
Nave 2019          0.1880 [-0.1237; 0.4996]       10.6      LOW:MODERATE
Park 2013i        -0.2891 [-1.1712; 0.5930]        5.9           LOW:LOW
Park 2013ii        0.0852 [-0.7918; 0.9622]        5.9           LOW:LOW
Park 2015          0.9514 [-0.0104; 1.9131]        5.3           LOW:LOW

Number of studies: k = 14
Number of observations: o = 642

                        SMD           95%-CI    t p-value
Random effects model 0.5118 [0.2036; 0.8199] 3.59  0.0033

Quantifying heterogeneity:
 tau^2 = 0.1929 [0.0277; 0.6167]; tau = 0.4392 [0.1664; 0.7853]
 I^2 = 60.1% [28.3%; 77.8%]; H = 1.58 [1.18; 2.12]

Quantifying residual heterogeneity:
 tau^2 = 0.1115; tau = 0.3339; I^2 = 11.1% [0.0%; 51.3%]; H = 1.06 [1.00; 1.43]

Test of heterogeneity:
     Q d.f. p-value
 32.58   13  0.0020

Results for subgroups (random effects model):
                                k     SMD             95%-CI  tau^2    tau    Q
Intensity = LOW:LOW             4  0.1698 [-0.6468; 0.9864] 0.1115 0.3339 3.72
Intensity = MODERATE:MODERATE   1 -0.0884 [-1.1827; 1.0059] 0.1115 0.3339 0.00
Intensity = LOW:MODERATE        5  0.3530 [-0.0773; 0.7833] 0.1115 0.3339 4.73
Intensity = LOW:HIGH            4  1.0667 [ 0.5401; 1.5933] 0.1115 0.3339 2.80
                                I^2
Intensity = LOW:LOW           19.4%
Intensity = MODERATE:MODERATE    --
Intensity = LOW:MODERATE      15.4%
Intensity = LOW:HIGH           0.0%

Test for subgroup differences (random effects model):
```

```
                  Q d.f. p-value
Between groups 14.78    3  0.0020
Within groups  11.25   10  0.3381

Details on meta-analytical method:
- Inverse variance method
- Sidik-Jonkman estimator for tau^2
  (assuming common tau^2 in subgroups)
- Q-Profile method for confidence interval of tau^2 and tau
- Hartung-Knapp adjustment for random effects model (df = 13)
- Hedges' g (bias corrected standardised mean difference; using exact formulae)
```

1.1.1.2 Amount

```
Warning: Redundant predictors dropped from the model.

                      SMD            95%-CI %W(random) amount.mins.delta.f
Aguiar 2020        0.6923 [-0.1731; 1.5577]        6.0               Equal
Brauer 2022        0.1479 [-0.2299; 0.5257]       10.1               Equal
Combs-Miller 2014 -0.0884 [-0.9654; 0.7887]        5.9               Equal
Gama 2017          0.0273 [-0.8085; 0.8631]        6.2               Equal
Hornby 2019i       1.0296 [ 0.4975; 1.5616]        8.7               Lower
Hornby 2019ii      0.9491 [ 0.4124; 1.4857]        8.6               Lower
Kang 2012i         0.1116 [-0.7657; 0.9890]        5.9               Equal
Kang 2012ii        1.0323 [ 0.0867; 1.9778]        5.4               Equal
MacKay-Lyons 2013  0.6377 [ 0.0372; 1.2382]        8.1               Equal
Macko 2005         1.5392 [ 0.8639; 2.2145]        7.4               Equal
Nave 2019          0.1880 [-0.1237; 0.4996]       10.6               Equal
Park 2013i        -0.2891 [-1.1712; 0.5930]        5.9               Equal
Park 2013ii        0.0852 [-0.7918; 0.9622]        5.9               Equal
Park 2015          0.9514 [-0.0104; 1.9131]        5.3               Equal

Number of studies: k = 14
Number of observations: o = 642

                        SMD           95%-CI    t p-value
Random effects model 0.5118 [0.2036; 0.8199] 3.59  0.0033

Quantifying heterogeneity:
 tau^2 = 0.1929 [0.0277; 0.6167]; tau = 0.4392 [0.1664; 0.7853]
 I^2 = 60.1% [28.3%; 77.8%]; H = 1.58 [1.18; 2.12]

Quantifying residual heterogeneity:
 tau^2 = 0.1729; tau = 0.4158; I^2 = 48.4% [2.0%; 72.8%]; H = 1.39 [1.01; 1.92]

Test of heterogeneity:
     Q d.f. p-value
 32.58   13  0.0020

Results for subgroups (random effects model):
                              k    SMD           95%-CI  tau^2    tau     Q
amount.mins.delta.f = Lower   2 0.9894 [0.4781; 1.5007] 0.1729 0.4158  0.04
amount.mins.delta.f = Equal  12 0.4106 [0.0719; 0.7492] 0.1729 0.4158 23.21
                              I^2
amount.mins.delta.f = Lower  0.0%
amount.mins.delta.f = Equal 52.6%

Test for subgroup differences (random effects model):
                   Q d.f. p-value
Between groups 13.25    1  0.0003
Within groups  23.25   12  0.0257

Details on meta-analytical method:
- Inverse variance method
- Sidik-Jonkman estimator for tau^2
  (assuming common tau^2 in subgroups)
```

```
- Q-Profile method for confidence interval of tau^2 and tau
- Hartung-Knapp adjustment for random effects model (df = 13)
- Hedges' g (bias corrected standardised mean difference; using exact formulae)
```

1.1.1.3 Dosage

```
##
## Mixed-Effects Model (k = 14; tau^2 estimator: SJ)
##
## tau^2 (estimated amount of residual heterogeneity):     0.1070 (SE = 0.0668)
## tau (square root of estimated tau^2 value):             0.3271
## I^2 (residual heterogeneity / unaccounted variability): 52.26%
## H^2 (unaccounted variability / sampling variability):   2.09
## R^2 (amount of heterogeneity accounted for):            44.53%
##
## Test for Residual Heterogeneity:
## QE(df = 12) = 16.0665, p-val = 0.1882
##
## Test of Moderators (coefficient 2):
## F(df1 = 1, df2 = 12) = 11.9581, p-val = 0.0047
##
## Model Results:
##
##          estimate      se    tval  df    pval    ci.lb   ci.ub
## intrcpt    0.1906  0.1392  1.3690  12  0.1961  -0.1127  0.4938
## Dosage     0.0131  0.0038  3.4580  12  0.0047   0.0049  0.0214  **
##
## ---
## Signif. codes:  0 '***' 0.001 '**' 0.01 '*' 0.05 '.' 0.1 ' ' 1
```

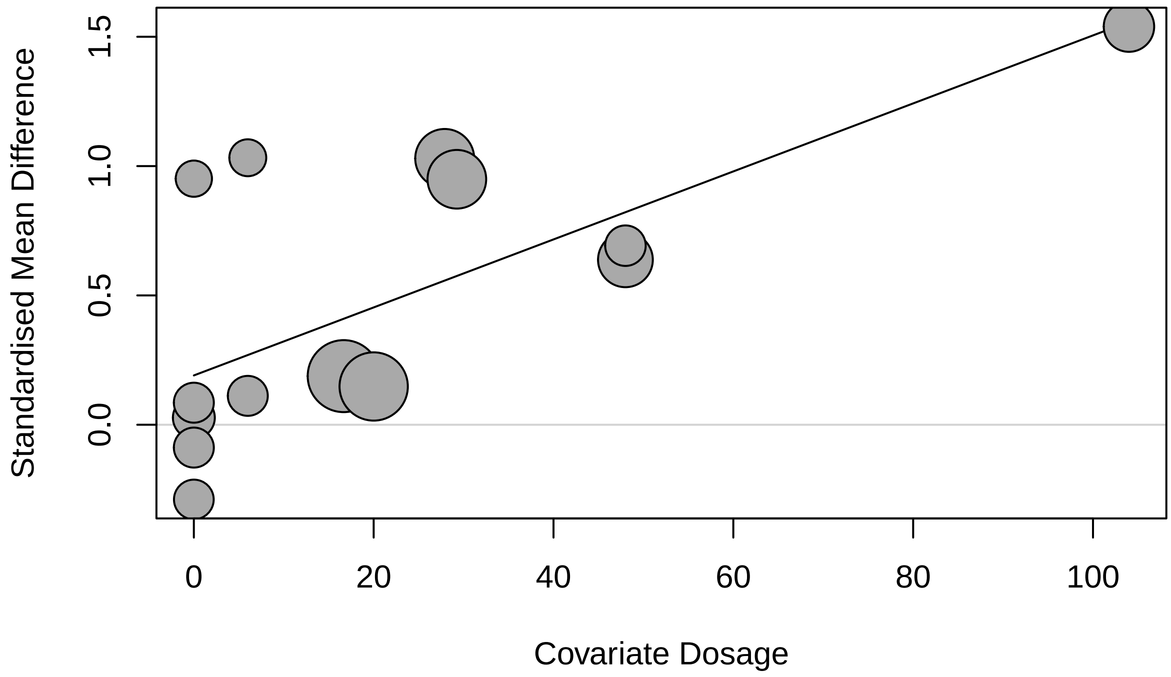

1.1.2 Walking Speed

1.1.2.1 Intensity

```
Warning in metacont(Exp.post.n, Exp.post.minus.pre.mean.sel,
Exp.post.minus.pre.sd.sel, : Note, studies with non-positive values for sd.e or
sd.c get no weight in meta-analysis.

                      SMD            95%-CI %W(random)         Intensity
Aguiar 2020       -0.3501 [-1.1935; 0.4933]        5.8          LOW:HIGH
Brauer 2022        0.1931 [-0.1851; 0.5712]       10.6      LOW:MODERATE
Combs-Miller 2014 -0.2282 [-1.1082; 0.6518]        5.5 MODERATE:MODERATE
Eich 2004          0.9756 [ 0.3867; 1.5646]        8.2          LOW:HIGH
Gama 2017         -0.0314 [-0.7723; 0.7094]        6.6           LOW:LOW
Kang 2012i         0.0000 [-0.8765; 0.8765]        5.5      LOW:MODERATE
Kang 2012ii        1.2113 [ 0.2410; 2.1817]        4.9      LOW:MODERATE
Kim & Yim 2017     0.7699 [ 0.0113; 1.5286]        6.5           LOW:LOW
Laufer 2001        0.3926 [-0.4009; 1.1860]        6.2           LOW:LOW
MacKay-Lyons 2013  0.3830 [-0.2073; 0.9733]        8.1      LOW:MODERATE
Nave 2019          0.2013 [-0.0984; 0.5010]       11.5      LOW:MODERATE
Park 2013i             NA                          0.0           LOW:LOW
Park 2013ii        0.0000 [-0.8765; 0.8765]        5.5           LOW:LOW
Pohl 2002          1.0978 [ 0.4285; 1.7671]        7.3          LOW:HIGH
Pohl 2002          0.4060 [-0.2208; 1.0328]        7.8          LOW:HIGH

Number of studies: k = 14
Number of observations: o = 659

                        SMD           95%-CI    t p-value
Random effects model 0.3647 [0.0996; 0.6298] 2.97  0.0108

Quantifying heterogeneity:
 tau^2 = 0.1394 [0.0000; 0.4592]; tau = 0.3734 [0.0000; 0.6776]
 I^2 = 39.5% [0.0%; 67.9%]; H = 1.29 [1.00; 1.76]

Quantifying residual heterogeneity:
 tau^2 = 0.1495; tau = 0.3866; I^2 = 37.8% [0.0%; 69.4%]; H = 1.27 [1.00; 1.81]

Test of heterogeneity:
     Q d.f. p-value
 21.50   13  0.0636

Results for subgroups (random effects model):
                                k     SMD            95%-CI  tau^2    tau    Q
Intensity = LOW:LOW             4  0.2901 [-0.3166; 0.8968] 0.1495 0.3866 2.74
Intensity = MODERATE:MODERATE   1 -0.2282 [-1.3895; 0.9331] 0.1495 0.3866 0.00
Intensity = LOW:MODERATE        5  0.3315 [-0.1497; 0.8128] 0.1495 0.3866 4.46
Intensity = LOW:HIGH            4  0.5885 [-0.4063; 1.5832] 0.1495 0.3866 8.87
                                I^2
Intensity = LOW:LOW            0.0%
Intensity = MODERATE:MODERATE    --
Intensity = LOW:MODERATE      10.4%
Intensity = LOW:HIGH          66.2%
```

```
Test for subgroup differences (random effects model):
                   Q d.f. p-value
Between groups  1.62    3  0.6560
Within groups  16.08   10  0.0973

Details on meta-analytical method:
- Inverse variance method
- Sidik-Jonkman estimator for tau^2
  (assuming common tau^2 in subgroups)
- Q-Profile method for confidence interval of tau^2 and tau
- Hartung-Knapp adjustment for random effects model (df = 13)
- Hedges' g (bias corrected standardised mean difference; using exact formulae)
```

1.1.2.2 Amount

```
                     SMD            95%-CI %W(random) amount.mins.delta.f
Aguiar 2020      -0.3501 [-1.1935; 0.4933]        5.8               Equal
Brauer 2022       0.1931 [-0.1851; 0.5712]       10.6               Equal
Combs-Miller 2014 -0.2282 [-1.1082; 0.6518]       5.5               Equal
Eich 2004         0.9756 [ 0.3867; 1.5646]        8.2               Equal
Gama 2017        -0.0314 [-0.7723; 0.7094]        6.6               Equal
Kang 2012i        0.0000 [-0.8765; 0.8765]        5.5               Equal
Kang 2012ii       1.2113 [ 0.2410; 2.1817]        4.9               Equal
Kim & Yim 2017    0.7699 [ 0.0113; 1.5286]        6.5              Higher
Laufer 2001       0.3926 [-0.4009; 1.1860]        6.2               Equal
MacKay-Lyons 2013  0.3830 [-0.2073; 0.9733]       8.1               Equal
Nave 2019         0.2013 [-0.0984; 0.5010]       11.5               Equal
Park 2013i            NA                          0.0               Equal
Park 2013ii       0.0000 [-0.8765; 0.8765]        5.5               Equal
Pohl 2002         1.0978 [ 0.4285; 1.7671]        7.3               Lower
Pohl 2002         0.4060 [-0.2208; 1.0328]        7.8               Lower

Number of studies: k = 14
Number of observations: o = 659

                        SMD           95%-CI    t p-value
Random effects model 0.3647 [0.0996; 0.6298] 2.97  0.0108

Quantifying heterogeneity:
 tau^2 = 0.1394 [0.0000; 0.4592]; tau = 0.3734 [0.0000; 0.6776]
 I^2 = 39.5% [0.0%; 67.9%]; H = 1.29 [1.00; 1.76]

Quantifying residual heterogeneity:
 tau^2 = 0.1307; tau = 0.3616; I^2 = 33.5% [0.0%; 66.5%]; H = 1.23 [1.00; 1.73]

Test of heterogeneity:
     Q d.f. p-value
 21.50   13  0.0636

Results for subgroups (random effects model):
                               k    SMD             95%-CI  tau^2    tau     Q
amount.mins.delta.f = Lower    2 0.7416 [-3.6513; 5.1345] 0.1307 0.3616  2.19
amount.mins.delta.f = Equal   11 0.2590 [-0.0342; 0.5522] 0.1307 0.3616 14.37
amount.mins.delta.f = Higher   1 0.7699 [-0.2682; 1.8081] 0.1307 0.3616  0.00
                               I^2
amount.mins.delta.f = Lower  54.3%
amount.mins.delta.f = Equal  30.4%
amount.mins.delta.f = Higher    --

Test for subgroup differences (random effects model):
                   Q d.f. p-value
Between groups  2.39    2  0.3033
Within groups  16.55   11  0.1219

Details on meta-analytical method:
- Inverse variance method
- Sidik-Jonkman estimator for tau^2
```

```
  (assuming common tau^2 in subgroups)
- Q-Profile method for confidence interval of tau^2 and tau
- Hartung-Knapp adjustment for random effects model (df = 13)
- Hedges' g (bias corrected standardised mean difference; using exact formulae)
```

1.1.2.3 Dosage

```
Warning: 1 study with NAs omitted from model fitting.


Mixed-Effects Model (k = 14; tau^2 estimator: SJ)

tau^2 (estimated amount of residual heterogeneity):     0.1436 (SE = 0.0707)
tau (square root of estimated tau^2 value):             0.3789
I^2 (residual heterogeneity / unaccounted variability): 59.73%
H^2 (unaccounted variability / sampling variability):   2.48
R^2 (amount of heterogeneity accounted for):            0.00%

Test for Residual Heterogeneity:
QE(df = 12) = 19.3736, p-val = 0.0799

Test of Moderators (coefficient 2):
F(df1 = 1, df2 = 12) = 0.7110, p-val = 0.4156

Model Results:

         estimate      se    tval  df    pval    ci.lb   ci.ub
intrcpt    0.2544  0.1804  1.4099  12  0.1840  -0.1387  0.6475
Dosage     0.0055  0.0065  0.8432  12  0.4156  -0.0087  0.0197

---
Signif. codes:  0 '***' 0.001 '**' 0.01 '*' 0.05 '.' 0.1 ' ' 1
```

1.2 Treadmill vs Treadmill

1.2.1 Walking Endurance

1.2.1.1 Intensity

```
                  SMD            95%-CI %W(random)         Intensity
Ada 2013         0.5730 [ 0.0837; 1.0622]       14.9 MODERATE:MODERATE
Alipsatici 2020  0.3188 [-0.4274; 1.0650]       12.9     MODERATE:HIGH
An 2020          2.2640 [ 1.4084; 3.1197]       12.0 MODERATE:MODERATE
Broderick 2019   0.0347 [-0.6810; 0.7504]       13.2           LOW:LOW
Kim & Kang 2018  0.8451 [ 0.0520; 1.6381]       12.5 MODERATE:MODERATE
Kim & Kim 2018a  1.0860 [ 0.1989; 1.9731]       11.8 MODERATE:MODERATE
Krzisnik 2021   -0.3841 [-1.2291; 0.4609]       12.1           LOW:LOW
Munari 2018      0.4607 [-0.5714; 1.4927]       10.6          LOW:HIGH

Number of studies: k = 8
Number of observations: o = 248

                        SMD            95%-CI    t p-value
Random effects model 0.6393 [-0.0164; 1.2950] 2.31  0.0545

Quantifying heterogeneity:
 tau^2 = 0.4754 [0.0937; 2.4389]; tau = 0.6895 [0.3060; 1.5617]
 I^2 = 71.2% [40.5%; 86.0%]; H = 1.86 [1.30; 2.68]

Quantifying residual heterogeneity:
 tau^2 = 0.3413; tau = 0.5842; I^2 = 66.7% [13.5%; 87.2%]; H = 1.73 [1.08; 2.80]

Test of heterogeneity:
     Q d.f. p-value
 24.28    7  0.0010

Results for subgroups (random effects model):
                                k     SMD            95%-CI  tau^2    tau     Q
Intensity = LOW:LOW             2 -0.1637 [-2.8208; 2.4934] 0.3413 0.5842  0.55
Intensity = MODERATE:MODERATE   4  1.1431 [-0.0334; 2.3197] 0.3413 0.5842 11.48
Intensity = LOW:HIGH            1  0.4607 [-1.0809; 2.0023] 0.3413 0.5842  0.00
Intensity = MODERATE:HIGH       1  0.3188 [-1.0480; 1.6856] 0.3413 0.5842  0.00
                                I^2
Intensity = LOW:LOW            0.0%
Intensity = MODERATE:MODERATE 73.9%
Intensity = LOW:HIGH             --
Intensity = MODERATE:HIGH        --

Test for subgroup differences (random effects model):
                   Q d.f. p-value
Between groups  9.66    3  0.0217
Within groups  12.03    4  0.0172

Details on meta-analytical method:
- Inverse variance method
- Sidik-Jonkman estimator for tau^2
  (assuming common tau^2 in subgroups)
```

```
- Q-Profile method for confidence interval of tau^2 and tau
- Hartung-Knapp adjustment for random effects model (df = 7)
- Hedges' g (bias corrected standardised mean difference; using exact formulae)
```

1.2.1.2 Amount

```
                    SMD            95%-CI %W(random) amount.mins.delta.f
Ada 2013         0.5730 [ 0.0837; 1.0622]       14.9              Higher
Alipsatici 2020  0.3188 [-0.4274; 1.0650]       12.9               Equal
An 2020          2.2640 [ 1.4084; 3.1197]       12.0               Equal
Broderick 2019   0.0347 [-0.6810; 0.7504]       13.2               Equal
Kim & Kang 2018  0.8451 [ 0.0520; 1.6381]       12.5               Equal
Kim & Kim 2018a  1.0860 [ 0.1989; 1.9731]       11.8               Equal
Krzisnik 2021   -0.3841 [-1.2291; 0.4609]       12.1               Equal
Munari 2018      0.4607 [-0.5714; 1.4927]       10.6               Equal

Number of studies: k = 8
Number of observations: o = 248

                        SMD            95%-CI    t p-value
Random effects model 0.6393 [-0.0164; 1.2950] 2.31  0.0545

Quantifying heterogeneity:
 tau^2 = 0.4754 [0.0937; 2.4389]; tau = 0.6895 [0.3060; 1.5617]
 I^2 = 71.2% [40.5%; 86.0%]; H = 1.86 [1.30; 2.68]

Quantifying residual heterogeneity:
 tau^2 = 0.5539; tau = 0.7442; I^2 = 75.3% [47.6%; 88.3%]; H = 2.01 [1.38; 2.93]

Test of heterogeneity:
     Q d.f. p-value
 24.28    7  0.0010

Results for subgroups (random effects model):
                               k    SMD            95%-CI  tau^2    tau     Q
amount.mins.delta.f = Equal    7 0.6521 [-0.1420; 1.4461] 0.5539 0.7442 24.26
amount.mins.delta.f = Higher   1 0.5730 [-0.9656; 2.1115] 0.5539 0.7442  0.00
                               I^2
amount.mins.delta.f = Equal  75.3%
amount.mins.delta.f = Higher    --

Test for subgroup differences (random effects model):
                   Q d.f. p-value
Between groups  0.01    1  0.9258
Within groups  24.26    6  0.0005

Details on meta-analytical method:
- Inverse variance method
- Sidik-Jonkman estimator for tau^2
  (assuming common tau^2 in subgroups)
- Q-Profile method for confidence interval of tau^2 and tau
- Hartung-Knapp adjustment for random effects model (df = 7)
- Hedges' g (bias corrected standardised mean difference; using exact formulae)
```

1.2.1.3 Dosage

```
Mixed-Effects Model (k = 8; tau^2 estimator: SJ)

tau^2 (estimated amount of residual heterogeneity):     0.5377 (SE = 0.3125)
tau (square root of estimated tau^2 value):             0.7333
I^2 (residual heterogeneity / unaccounted variability): 78.71%
H^2 (unaccounted variability / sampling variability):   4.70
R^2 (amount of heterogeneity accounted for):            0.00%

Test for Residual Heterogeneity:
QE(df = 6) = 23.7011, p-val = 0.0006

Test of Moderators (coefficient 2):
F(df1 = 1, df2 = 6) = 0.1895, p-val = 0.6786

Model Results:

         estimate      se     tval  df    pval    ci.lb   ci.ub
intrcpt    0.7272  0.3565   2.0397   6  0.0875  -0.1452  1.5996  .
Dosage    -0.0060  0.0138  -0.4353   6  0.6786  -0.0397  0.0277

---
Signif. codes:  0 '***' 0.001 '**' 0.01 '*' 0.05 '.' 0.1 ' ' 1
```

1.2.2 Walking Speed

1.2.2.1 Intensity

```
                        SMD            95%-CI %W(random)         Intensity
Ada 2013             0.3827 [-0.1009; 0.8663]       12.7 MODERATE:MODERATE
Alipsatici 2020      0.6910 [-0.0749; 1.4569]        8.9     MODERATE:HIGH
An 2020              1.0238 [ 0.3243; 1.7232]        9.7 MODERATE:MODERATE
Broderick 2019       0.0449 [-0.6708; 0.7607]        9.5           LOW:LOW
Druzbicki 2018       1.1273 [ 0.3494; 1.9052]        8.7           LOW:LOW
Kim & Kang 2018      0.9783 [ 0.1727; 1.7838]        8.4 MODERATE:MODERATE
Kim & Kim 2018a      0.9646 [ 0.0916; 1.8376]        7.7 MODERATE:MODERATE
Kim & Kim 2018b      1.5920 [ 0.6922; 2.4918]        7.4           LOW:LOW
Krzisnik 2021       -0.0819 [-0.9180; 0.7543]        8.1           LOW:LOW
Munari 2018          0.5922 [-0.4512; 1.6356]        6.2          LOW:HIGH
Park & Chung 2018i   1.4671 [ 0.4270; 2.5072]        6.2           LOW:LOW
Park & Chung 2018ii  0.9262 [-0.0637; 1.9160]        6.6           LOW:LOW

Number of studies: k = 12
Number of observations: o = 341

                        SMD           95%-CI    t p-value
Random effects model 0.7682 [0.4460; 1.0905] 5.25  0.0003

Quantifying heterogeneity:
 tau^2 = 0.1499 [0.0000; 0.5726]; tau = 0.3871 [0.0000; 0.7567]
 I^2 = 35.7% [0.0%; 67.5%]; H = 1.25 [1.00; 1.76]

Quantifying residual heterogeneity:
 tau^2 = 0.2031; tau = 0.4507; I^2 = 53.0% [0.1%; 77.9%]; H = 1.46 [1.00; 2.13]

Test of heterogeneity:
     Q d.f. p-value
 17.11   11  0.1046

Results for subgroups (random effects model):
                                k    SMD            95%-CI  tau^2    tau     Q
Intensity = LOW:LOW             6 0.7959 [ 0.0405; 1.5514] 0.2031 0.4507 13.74
Intensity = MODERATE:MODERATE   4 0.7988 [ 0.2778; 1.3198] 0.2031 0.4507  3.29
Intensity = LOW:HIGH            1 0.5922 [-0.7749; 1.9593] 0.2031 0.4507  0.00
Intensity = MODERATE:HIGH       1 0.6910 [-0.4781; 1.8602] 0.2031 0.4507  0.00
                                I^2
Intensity = LOW:LOW           63.6%
Intensity = MODERATE:MODERATE  8.7%
Intensity = LOW:HIGH             --
Intensity = MODERATE:HIGH        --

Test for subgroup differences (random effects model):
                   Q d.f. p-value
Between groups  0.11    3  0.9906
Within groups  17.03    8  0.0298

Details on meta-analytical method:
- Inverse variance method
```

```
- Sidik-Jonkman estimator for tau^2
  (assuming common tau^2 in subgroups)
- Q-Profile method for confidence interval of tau^2 and tau
- Hartung-Knapp adjustment for random effects model (df = 11)
- Hedges' g (bias corrected standardised mean difference; using exact formulae)
```

1.2.2.2 Amount

```
                        SMD            95%-CI %W(random) amount.mins.delta.f
Ada 2013             0.3827 [-0.1009; 0.8663]       12.7              Higher
Alipsatici 2020      0.6910 [-0.0749; 1.4569]        8.9               Equal
An 2020              1.0238 [ 0.3243; 1.7232]        9.7               Equal
Broderick 2019       0.0449 [-0.6708; 0.7607]        9.5               Equal
Druzbicki 2018       1.1273 [ 0.3494; 1.9052]        8.7               Equal
Kim & Kang 2018      0.9783 [ 0.1727; 1.7838]        8.4               Equal
Kim & Kim 2018a      0.9646 [ 0.0916; 1.8376]        7.7               Equal
Kim & Kim 2018b      1.5920 [ 0.6922; 2.4918]        7.4               Equal
Krzisnik 2021       -0.0819 [-0.9180; 0.7543]        8.1               Equal
Munari 2018          0.5922 [-0.4512; 1.6356]        6.2               Equal
Park & Chung 2018i   1.4671 [ 0.4270; 2.5072]        6.2               Equal
Park & Chung 2018ii  0.9262 [-0.0637; 1.9160]        6.6               Equal

Number of studies: k = 12
Number of observations: o = 341

                        SMD           95%-CI    t p-value
Random effects model 0.7682 [0.4460; 1.0905] 5.25  0.0003

Quantifying heterogeneity:
 tau^2 = 0.1499 [0.0000; 0.5726]; tau = 0.3871 [0.0000; 0.7567]
 I^2 = 35.7% [0.0%; 67.5%]; H = 1.25 [1.00; 1.76]

Quantifying residual heterogeneity:
 tau^2 = 0.1508; tau = 0.3883; I^2 = 32.4% [0.0%; 66.8%]; H = 1.22 [1.00; 1.73]

Test of heterogeneity:
     Q d.f. p-value
 17.11   11  0.1046

Results for subgroups (random effects model):
                               k    SMD            95%-CI  tau^2    tau     Q
amount.mins.delta.f = Equal   11 0.8244 [ 0.4755; 1.1734] 0.1508 0.3883 14.79
amount.mins.delta.f = Higher   1 0.3827 [-0.5190; 1.2844] 0.1508 0.3883  0.00
                               I^2
amount.mins.delta.f = Equal  32.4%
amount.mins.delta.f = Higher    --

Test for subgroup differences (random effects model):
                   Q d.f. p-value
Between groups  0.83    1  0.3634
Within groups  14.79   10  0.1398

Details on meta-analytical method:
- Inverse variance method
- Sidik-Jonkman estimator for tau^2
  (assuming common tau^2 in subgroups)
- Q-Profile method for confidence interval of tau^2 and tau
- Hartung-Knapp adjustment for random effects model (df = 11)
- Hedges' g (bias corrected standardised mean difference; using exact formulae)
```

1.2.2.3 Dosage

```
Mixed-Effects Model (k = 12; tau^2 estimator: SJ)

tau^2 (estimated amount of residual heterogeneity):     0.1556 (SE = 0.0854)
tau (square root of estimated tau^2 value):             0.3944
I^2 (residual heterogeneity / unaccounted variability): 49.31%
H^2 (unaccounted variability / sampling variability):   1.97
R^2 (amount of heterogeneity accounted for):            0.00%

Test for Residual Heterogeneity:
QE(df = 10) = 15.8672, p-val = 0.1035

Test of Moderators (coefficient 2):
F(df1 = 1, df2 = 10) = 0.6067, p-val = 0.4541

Model Results:

         estimate      se     tval  df    pval    ci.lb   ci.ub
intrcpt    0.8318  0.1695   4.9067  10  0.0006   0.4541  1.2096  ***
Dosage    -0.0064  0.0082  -0.7789  10  0.4541  -0.0248  0.0119

---
Signif. codes:  0 '***' 0.001 '**' 0.01 '*' 0.05 '.' 0.1 ' ' 1
```

References

Ada, L., Dean, C.M., Lindley, R., 2013. Randomized trial of treadmill training to improve walking in community-dwelling people after stroke: the AMBULATE trial. Int J Stroke 8, 436-444. doi:10.1111/j.1747-4949.2012.00934.x

Aguiar, L.T., Nadeau, S., Britto, R.R., Teixeira-Salmela, L.F., Martins, J.C., Samora, G.A.R., da Silva Junior, J.A., Faria, C., 2020. Effects of aerobic training on physical activity in people with stroke: A randomized controlled trial. NeuroRehabilitation 46, 391-401. doi:10.3233/NRE-193013

Alipsatici, C., Alaca, N., Canbora, M., 2020. Comparison of the effects of treadmill trainings on walking and balance functions by increasing the speed and incline in chronic patients with stroke. Turk J Neurol 26, 316-321. doi:10.4274/tnd.2020.48921

An, B., Woo, Y., Park, K., Kim, S., 2020. Effects of insole on the less affected side during execution of treadmill walking training on gait ability in chronic stroke patients: A preliminary study. Restor Neurol Neurosci 38, 375-384. doi:10.3233/RNN-201040

Brauer, S.G., Kuys, S.S., Ada, L., Paratz, J.D., 2022. Improving Physical Activity after stroke via Treadmill training (IMPACT) and self-management: A randomized trial. Int J Stroke 17, 1137-1144. doi:10.1177/17474930221078121

Broderick, P., Horgan, F., Blake, C., Ehrensberger, M., Simpson, D., Monaghan, K., 2019. Mirror therapy and treadmill training for patients with chronic stroke: a pilot randomized controlled trial. Top Stroke Rehabil 26, 163-172. doi:10.1080/10749357.2018.1556504

Combs-Miller, S.A., Kalpathi Parameswaran, A., Colburn, D., Ertel, T., Harmeyer, A., Tucker, L., Schmid, A.A., 2014. Body weight-supported treadmill training vs. overground walking training for persons with chronic stroke: a pilot randomized controlled trial. Clin Rehabil 28, 873-884. doi:10.1177/0269215514520773

Druzbicki, M., Przysada, G., Guzik, A., Brzozowska-Magon, A., Kolodziej, K., Wolan-Nieroda, A., Majewska, J., Kwolek, A., 2018. The Efficacy of Gait Training Using a Body Weight Support Treadmill and Visual Biofeedback in Patients with Subacute Stroke: A Randomized Controlled Trial. Biomed Res Int 2018, 3812602. doi:10.1155/2018/3812602

Eich, H.J., Mach, H., Werner, C., Hesse, S., 2004. Aerobic treadmill plus Bobath walking training improves walking in subacute stroke: A randomized controlled trial. Clin Rehabil 18, 640-651. doi:10.1191/0269215504cr779oa

Gama, G.L., Celestino, M.L., Barela, J.A., Forrester, L., Whitall, J., Barela, A.M., 2017. Effects of Gait Training With Body Weight Support on a Treadmill Versus Overground in Individuals With Stroke. Arch Phys Med Rehabil 98, 738-745. doi:10.1016/j.apmr.2016.11.022

Hornby, T.G., Henderson, C.E., Plawecki, A., Lucas, E., Lotter, J., Holthus, M., Brazg, G., Fahey, M., Woodward, J., Ardestani, M., Roth, E.J., 2019. Contributions of Stepping Intensity and Variability to Mobility in Individuals Poststroke. Stroke 50, 2492-2499. doi:10.1161/STROKEAHA.119.026254

Kang, H.K., Kim, Y., Chung, Y., Hwang, S., 2012. Effects of treadmill training with optic flow on balance and gait in individuals following stroke: randomized controlled trials. Clin Rehabil 26, 246-255. doi:10.1177/0269215511419383

Kim, B.R., Kang, T.W., 2018. The effects of proprioceptive neuromuscular facilitation lower-leg taping and treadmill training on mobility in patients with stroke. Int J Rehabil Res 41, 343-348. doi:10.1097/MRR.0000000000000309

Kim, C.H., Kim, Y.N., 2018a. Effects of proprioceptive neuromuscular facilitation and treadmill training on the balance and walking ability of stroke patients. J Korean Phys Ther 30, 79-83. doi:10.18857/jkpt.2018.30.3.79

Kim, J., Yim, J., 2017. Effects of an Exercise Protocol for Improving Handgrip Strength and Walking Speed on Cognitive Function in Patients with Chronic Stroke. Med Sci Monit 23, 5402-5409. doi:10.12659/msm.904723

Kim, K.J., Kim, K.H., 2018b. Progressive treadmill cognitive dual-task gait training on the gait ability in patients with chronic stroke. J Exerc Rehabil 14, 821-828. doi:10.12965/jer.1836370.185

Kržišnik, M., Horvat Rauter, B., Bizovčar, N., 2021. Effects of virtual reality-based treadmill training on the balance and gait ability in patients after stroke. Hrvatska revija za rehabilitacijska istraživanja 57, 92-102. doi:10.31299/hrri.57.2.6

Laufer, Y., Dickstein, R., Chefez, Y., Marcovitz, E., 2001. The effect of treadmill training on the ambulation of stroke survivors in the early stages of rehabilitation: A randomized study. J Rehab Res and Dev 38, 69-78.

Mackay-Lyons, M., McDonald, A., Matheson, J., Eskes, G., Klus, M.A., 2013. Dual effects of body-weight supported treadmill training on cardiovascular fitness and walking ability early after stroke: a randomized controlled trial. Neurorehabil Neural Repair 27, 644-653. doi:10.1177/1545968313484809

Macko, R.F., Ivey, F.M., Forrester, L.W., Hanley, D., Sorkin, J.D., Katzel, L.I., Silver, K.H., Goldberg, A.P., 2005. Treadmill exercise rehabilitation improves ambulatory function and cardiovascular fitness in patients with chronic stroke: a randomized, controlled trial. Stroke 36, 2206-2211. doi:10.1161/01.STR.0000181076.91805.89

Munari, D., Pedrinolla, A., Smania, N., Picelli, A., Gandolfi, M., Saltuari, L., Schena, F., 2018. High-intensity treadmill training improves gait ability, VO2peak and cost of walking in stroke survivors: preliminary results of a pilot randomized controlled trial. Eur J Phys Rehabil Med 54, 408-418. doi:10.23736/S1973-9087.16.04224-6

Nave, A.H., Rackoll, T., Grittner, U., Blasing, H., Gorsler, A., Nabavi, D.G., Audebert, H.J., Klostermann, F., Muller-Werdan, U., Steinhagen-Thiessen, E., Meisel, A., Endres, M., Hesse, S., Ebinger, M., Floel, A., 2019. Physical Fitness Training in Patients with Subacute Stroke (PHYS-STROKE): multicentre, randomised controlled, endpoint blinded trial. BMJ 366, l5101. doi:10.1136/bmj.l5101

Park, I.M., Lee, Y.S., Moon, B.M., Sim, S.M., 2013. A comparison of the effects of overground gait training and treadmill gait training according to stroke patients' gait velocity. J Phys Ther Sci 25, 379-382. doi:10.1589/jpts.25.379

Park, J., Park, S.Y., Kim, Y.W., Woo, Y., 2015. Comparison between treadmill training with rhythmic auditory stimulation and ground walking with rhythmic auditory stimulation on gait ability in chronic stroke patients: A pilot study. NeuroRehabilitation 37, 193-202. doi:10.3233/NRE-151252

Park, J.H., Chung, Y.J., 2018. Comparison of Aquatic Treadmill and Anti-Gravity Treadmill Gait Training to Improve Balance and Gait Abilities in Stroke Patients. The Journal of Korean Physical Therapy 30, 67-72. doi:10.18857/jkpt.2018.30.2.67

Pohl, M., Ritschel, C., Ruckriem, S., 2002. Speed-dependent treadmill training in ambulatory hemiparetic stroke patients A randomized controlled trial. Stroke 33, 553-558. doi:10.1161/hs0202.102365
