# Supplementary material for: Challenge Level Contributes to the Efficacy of Treadmill Interventions after Stroke: A Systematic Review and Meta-Analysis
Source: Brain Sci. 2023 Dec 18;13(12):1729. doi: 10.3390/brainsci13121729 (PMC10741427; doi:10.3390/brainsci13121729)
Supplement: Supplementary file 1 [file brainsci-13-01729-s001.zip › Supplementary Table S1. Intervention amount_revised.pdf]

Supplementary Table S1. Intervention Amount.

	Experimental Intervention					Comparison Intervention				
**Author**	**Duration (mins)**	**Frequency (per week)**	**Programme length (weeks)**	**Work Duration (mins)**	**Total Amount (mins)**	**Duration (mins)**	**Frequency (per week)**	**Programme length (weeks)**	**Work Duration (mins)**	**Total Amount (mins)**
**TT versus other physiotherapy interventions**										
Aguiar et al. (2020) [1]	40	3	12	40	1440	40	3	12	40	1440
Brauer et al. (2022) [2]	TT & self management or conv GT 30	TT:3<br>Conv GT:2	8	NR	1200	30	5	8	NR	1200
Combs-Miller et al. (2014) [3]	30	5	2	30	300	30	5	2	30	300
Eich et al. (2004) [4]	60	5	6	NR	1800	60	5	6	NR	1800
Gama et al. (2017) [5]	45	3	6	NR	810	45	3	6	NR	810
Hornby et al. (2019) [6]	EXP 1&2: 60	EXP 1&2: 3 to 5 (mean 3.375)	EXP 1&2: 8	EXP 1: 33<br>EXP 2: 34	EXP 1: 891<br>EXP 2: 918	60	3 to 5	8	37	999
Kang et al. (2012) [7]	EXP 1&2: 30	EXP 1&2: 3<br>Plus CVT:5	EXP 1&2: 4	EXP 1 & 2: 30	EXP 1&2: 360	30	3	4	NR	360
Kim & Yim (2017) [8]	TT: 20<br>Hand grip strength (HGS): 15<br>Conv rehab: 2 x 60	HGS+TT:3<br>Conv rehab:5	6	TT: 20	4230	2 x 60	5	6	NR	3600
Laufer et al. (2001) [9]	TT = 4 mins week 1, 6 mins week 2, 8 mins week 3	5	3	TT = 4 mins week 1, 6 mins week 2, 8 mins week 3	90	OGT = 4 mins week 1, 6 mins week 2, 8 mins week 3	5	3	OGT = 4 mins week 1, 6 mins week 2, 8 mins week 3	90
MacKay-Lyons et al. (2013) [10]	60	5 for 1st 6w, 3 for 2nd 6w	12	NR	2880	60	5 for 1st 6w, 3 for 2nd 6w	12	NR	2880
Macko et al. (2005) [11]	40	3	26	NR	3120	40	3	26	40	3120
Nave et al. (2019) [12]	50	5	4	TT: 21<br>Other: NR	1000	50	5	4	Relaxation 24<br>Other NR	1000
Park et al. (2013) [13]	30	10 (twice per day for 5 days)	EXP 1&2: 1	NR	300	30	10 (twice per day for 5 days)	1	NR	300

Park et al. (2015) [14]	30 TT (Conv rehab NR)	5	3	NR	450	30 OGT (Conv rehab NR)	5	3	NR	450
Pohl et al. (2002) [15]	EXP 1: TT 30 + Conv PT 45<br>EXP 2: TT 30 + Conv PT 45	TT: 3<br>Conv PT : 2	4	NR	EXP 1&2: 720	Conv GT:45<br>Conv PT:45	Conv GT: 3<br>Conv PT: 2	4	NR	900
**TT versus another type of TT**										
Ada et al. (2013) [16]	30	3	16	30	1440	COMP 1: 30<br>COMP 2: NA	COMP 1: 3<br>COMP 2: NA	COMP 1: 8<br>COMP 2: NA	COMP 1: 30	COMP 1: 720<br>COMP 2: NA
Alipsatici et al. (2020) [17]	Total 75<br>(TT 30, e-stim 15, conv rehab 30)	3	8	TT: 30	1800	75<br>(TT 30, e-stim 15, conv rehab 30)	3	8	TT: 30	1800
An et al. (2020) [18]	30	5	4	30	600	30	5	4	30	600
Broderick et al. (2019) [19]	30	3	4	30	360	30	3	4	30	360
Drużbicki et al. (2018) [20]	TT: 30<br>Conv rehab: 120 mins weekdays + 45 mins Saturdays	TT: 5<br>Conv: 6	3	TT: 30	2385	TT: 30<br>Conv rehab: 120 mins weekdays + 45 mins Saturdays	5	3	TT: 30	2385
Kim & Kang (2018) [21]	50	5	6	30	900	50	5	6	30	900
Kim & Kim (2018a) [22]	40 (PNF warmup 5, PNF 15 mins, TT 15 mins, PNF cooldown 5)	5	6	NR	1200	40 (PNF warmup 5, treadmill 30, PNF cooldown 5)	5	6	NR	1200
Kim & Kim (2018b) [23]	30	5	4	NR	600	30	5	4	NR	600
Kržišnik et al. (2021) [24]	Conv rehab: 90<br>TT: 15.5 (13mins for 2w + 18mins for 2w)	5	4	TT: 15.5 (13mins for 2w + 18mins for 2w)	2110	Conv rehab: 90<br>TT: 15.5	5	4	TT: 15.5 (13mins for 2w + 18mins for 2w)	2110
Munari et al. (2018) [25]	55	3	12	55	1980	55	3	12	55	1980
Park & Chung (2018) [26]	EXP 1 + 2: TT: 30<br>Conv PT: 30	EXP 1 + 2: TT: 3<br>Conv PT: 5	4	NR	EXP 1 +2: 960	30	TT: 3<br>Conv PT: 5	4	NR	960

Abbreviations: COMP, comparison; Conv, conventional; e-stim, electrical stimulation; EXP, experimental; GT, gait training; OGT, overground gait training; PNF, proprioceptive neuromuscular facilitation; PT, physiotherapy; NR, not reported; TT, treadmill training.

References

1. Aguiar, L.T.; Nadeau, S.; Britto, R.R.; Teixeira-Salmela, L.F.; Martins, J.C.; Samora, G.A.R.; da Silva Junior, J.A.; Faria, C. Effects of aerobic training on physical activity in people with stroke: A randomized controlled trial. *NeuroRehabilitation* **2020**, 46, 391-401; doi: 10.3233/NRE-193013.
2. Brauer, S.G.; Kuys, S.S.; Ada, L.; Paratz, J.D. Improving Physical Activity after stroke via Treadmill training (IMPACT) and self-management: A randomized trial. *Int J Stroke* **2022**, 17, 1137-44; doi: 10.1177/17474930221078121.
3. Combs-Miller, S.A.; Kalpathi Parameswaran, A.; Colburn, D.; Ertel, T.; Harmeyer, A.; Tucker, L.; Schmid, A.A. Body weight-supported treadmill training vs. overground walking training for persons with chronic stroke: a pilot randomized controlled trial. *Clin Rehabil* **2014**, 28, 873-84; doi: 10.1177/0269215514520773.
4. Eich, H.J.; Mach, H.; Werner, C.; Hesse, S. Aerobic treadmill plus Bobath walking training improves walking in subacute stroke: A randomized controlled trial. *Clin Rehabil* **2004**, 18, 640-51; doi: 10.1191/0269215504cr779oa.
5. Gama, G.L.; Celestino, M.L.; Barela, J.A.; Forrester, L.; Whitall, J.; Barela, A.M. Effects of Gait Training With Body Weight Support on a Treadmill Versus Overground in Individuals With Stroke. *Arch Phys Med Rehabil* **2017**, 98, 738-45; doi: 10.1016/j.apmr.2016.11.022.
6. Hornby, T.G.; Henderson, C.E.; Plawecki, A.; Lucas, E.; Lotter, J.; Holthus, M.; Brazg, G.; Fahey, M.; Woodward, J.; Ardestani, M.; Roth, E.J. Contributions of Stepping Intensity and Variability to Mobility in Individuals Poststroke. *Stroke* **2019**, 50, 2492-99; doi: 10.1161/STROKEAHA.119.026254.
7. Kang, H.K.; Kim, Y.; Chung, Y.; Hwang, S. Effects of treadmill training with optic flow on balance and gait in individuals following stroke: randomized controlled trials. *Clin Rehabil* **2012**, 26, 246-55; doi: 10.1177/0269215511419383.
8. Kim, J.; Yim, J. Effects of an Exercise Protocol for Improving Handgrip Strength and Walking Speed on Cognitive Function in Patients with Chronic Stroke. *Med Sci Monit* **2017**, 23, 5402-09; doi: 10.12659/msm.904723.
9. Laufer, Y.; Dickstein, R.; Chefez, Y.; Marcovitz, E. The effect of treadmill training on the ambulation of stroke survivors in the early stages of rehabilitation: A randomized study. *J Rehab Res and Dev* **2001**, 38, 69-78; doi:
10. Mackay-Lyons, M.; McDonald, A.; Matheson, J.; Eskes, G.; Klus, M.A. Dual effects of body-weight supported treadmill training on cardiovascular fitness and walking ability early after stroke: a randomized controlled trial. *Neurorehabil Neural Repair* **2013**, 27, 644-53; doi: 10.1177/1545968313484809.
11. Macko, R.F.; Ivey, F.M.; Forrester, L.W.; Hanley, D.; Sorkin, J.D.; Katzel, L.I.; Silver, K.H.; Goldberg, A.P. Treadmill exercise rehabilitation improves ambulatory function and cardiovascular fitness in patients with chronic stroke: a randomized, controlled trial. *Stroke* **2005**, 36, 2206-11; doi: 10.1161/01.STR.0000181076.91805.89.
12. Nave, A.H.; Rackoll, T.; Grittner, U.; Blasing, H.; Gorsler, A.; Nabavi, D.G.; Audebert, H.J.; Klostermann, F.; Muller-Werdan, U.; Steinhagen-Thiessen, E.; Meisel, A.; Endres, M.; Hesse, S.; Ebinger, M.; Floel, A. Physical Fitness Training in Patients with Subacute Stroke (PHYS-STROKE): multicentre, randomised controlled, endpoint blinded trial. *BMJ* **2019**, 366, l5101; doi: 10.1136/bmj.l5101.
13. Park, I.M.; Lee, Y.S.; Moon, B.M.; Sim, S.M. A comparison of the effects of overground gait training and treadmill gait training according to stroke patients' gait velocity. *J Phys Ther Sci* **2013**, 25, 379-82; doi: 10.1589/jpts.25.379.
14. Park, J.; Park, S.Y.; Kim, Y.W.; Woo, Y. Comparison between treadmill training with rhythmic auditory stimulation and ground walking with rhythmic auditory stimulation on gait ability in chronic stroke patients: A pilot study. *NeuroRehabilitation* **2015**, 37, 193-202; doi: 10.3233/NRE-151252.
15. Pohl, M.; Ritschel, C.; Ruckriem, S. Speed-dependent treadmill training in ambulatory hemiparetic stroke patients A randomized controlled trial. *Stroke* **2002**, 33, 553-58; doi: 10.1161/hs0202.102365.
16. Ada, L.; Dean, C.M.; Lindley, R. Randomized trial of treadmill training to improve walking in community-dwelling people after stroke: the AMBULATE trial. *Int J Stroke* **2013**, 8, 436-44; doi: 10.1111/j.1747-4949.2012.00934.x.

17. Alipsatici, C.; Alaca, N.; Canbora, M. Comparison of the effects of treadmill trainings on walking and balance functions by increasing the speed and incline in chronic patients with stroke. *Turk J Neurol* **2020**, 26, 316-21; doi: 10.4274/tnd.2020.48921.
18. An, B.; Woo, Y.; Park, K.; Kim, S. Effects of insole on the less affected side during execution of treadmill walking training on gait ability in chronic stroke patients: A preliminary study. *Restor Neurol Neurosci* **2020**, 38, 375-84; doi: 10.3233/RNN-201040.
19. Broderick, P.; Horgan, F.; Blake, C.; Ehrensberger, M.; Simpson, D.; Monaghan, K. Mirror therapy and treadmill training for patients with chronic stroke: a pilot randomized controlled trial. *Top Stroke Rehabil* **2019**, 26, 163-72; doi: 10.1080/10749357.2018.1556504.
20. Druzbicki, M.; Przysada, G.; Guzik, A.; Brzozowska-Magon, A.; Kolodziej, K.; Wolan-Nieroda, A.; Majewska, J.; Kwolek, A. The Efficacy of Gait Training Using a Body Weight Support Treadmill and Visual Biofeedback in Patients with Subacute Stroke: A Randomized Controlled Trial. *Biomed Res Int* **2018**, 2018, 3812602; doi: 10.1155/2018/3812602.
21. Kim, B.R.; Kang, T.W. The effects of proprioceptive neuromuscular facilitation lower-leg taping and treadmill training on mobility in patients with stroke. *Int J Rehabil Res* **2018**, 41, 343-48; doi: 10.1097/MRR.0000000000000309.
22. Kim, C.-H.; Kim, Y.-N. Effects of proprioceptive neuromuscular facilitation and treadmill training on the balance and walking ability of stroke patients. *J Korean Phys Ther* **2018a**, 30, 79-83; doi: 10.18857/jkpt.2018.30.3.79.
23. Kim, K.J.; Kim, K.H. Progressive treadmill cognitive dual-task gait training on the gait ability in patients with chronic stroke. *J Exerc Rehabil* **2018b**, 14, 821-28; doi: 10.12965/jer.1836370.185.
24. Kržišnik, M.; Horvat Rauter, B.; Bizovčar, N. Effects of virtual reality-based treadmill training on the balance and gait ability in patients after stroke. *Hrvatska revija za rehabilitacijska istraživanja* **2021**, 57, 92-102; doi: 10.31299/hrri.57.2.6.
25. Munari, D.; Pedrinolla, A.; Smania, N.; Picelli, A.; Gandolfi, M.; Saltuari, L.; Schena, F. High-intensity treadmill training improves gait ability, VO2peak and cost of walking in stroke survivors: preliminary results of a pilot randomized controlled trial. *Eur J Phys Rehabil Med* **2018**, 54, 408-18; doi: 10.23736/S1973-9087.16.04224-6.
26. Park, J.H.; Chung, Y.J. Comparison of Aquatic Treadmill and Anti-Gravity Treadmill Gait Training to Improve Balance and Gait Abilities in Stroke Patients. *The Journal of Korean Physical Therapy* **2018**, 30, 67-72; doi: 10.18857/jkpt.2018.30.2.67.
